# Catenanes: Fifty Years of Molecular Links

**DOI:** 10.1002/anie.201411619

**Published:** 2015-05-07

**Authors:** Guzmán Gil-Ramírez, David A Leigh, Alexander J Stephens

**Affiliations:** School of Chemistry, University of ManchesterOxford Road, Manchester, M13 9PL (UK)

**Keywords:** catenanes, interlocked molecules, links, supramolecular chemistry, template synthesis

## Abstract

Half a century after Schill and Lüttringhaus carried out the first directed synthesis of a [2]catenane, a plethora of strategies now exist for the construction of molecular Hopf links (singly interlocked rings), the simplest type of catenane. The precision and effectiveness with which suitable templates and/or noncovalent interactions can arrange building blocks has also enabled the synthesis of intricate and often beautiful higher order interlocked systems, including Solomon links, Borromean rings, and a Star of David catenane. This Review outlines the diverse strategies that exist for synthesizing catenanes in the 21st century and examines their emerging applications and the challenges that still exist for the synthesis of more complex topologies.

## 1. Introduction

Over the last fifty years, research into the synthesis of interlocked molecules has evolved from a concept viewed with some scepticism to a reality in which ways to harness the properties afforded by mechanical bonding are now being sought. Catenanes are at the forefront of efforts to make artificial molecular machines and to exploit the dynamics of interlocked structures in polymers, MOFs, and other materials. Their study has led to significant developments that have implications not only in supramolecular chemistry but across a range of scientific disciplines from biology to soft matter physics. Here we review the synthetic tactics that have been employed in the formation of catenanes, including the various template techniques and methods used in the contextual synthesis of singly interlocked [2]catenanes (Section 2), strategies for higher order interlocked structures (Sections 3 and 4), the properties of catenanes (Section 5), and their incorporation into materials and onto surfaces (Section 6). The vast literature featuring interlocked molecular rings means that only examples of the most significant advances of the last fifty years could be covered. All of the cap-and-stick structures shown in the Review are original representations produced from coordinates taken from the Cambridge Structural Database (CSD).

### 1.1. Historical Background

**Scheme 1:**
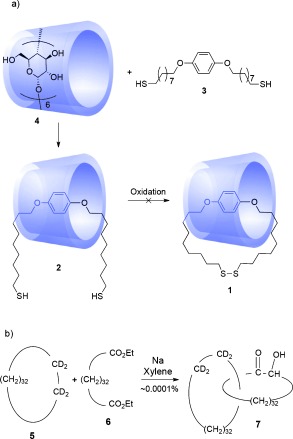
a) Attempted synthesis of a [2]catenane by Lüttringhaus and Cramer (1958). Complexation between dithiol 3 and α-cyclodextrin 4 led to a threaded inclusion complex (2), but subsequent oxidation did not afford catenane 1.[11] b) Wasserman's 1960 synthesis of a [2]catenane (7) by statistical threading of diester 6 through macrocycle 5 during an acyloin condensation.[1]

The earliest known mention of the possibility of mechanically linked cyclic molecules, structures later termed “catenanes”[1] is attributed[2] to the 1915 Nobel Laureate in Chemistry, Richard Willstätter, in a seminar in Zürich some time in the period 1906–1912. Unfortunately, the nature of that discussion is lost in the mists of time, but it is remarkable that the notion of interlocked molecules was raised at all in an era that predated the assertion of Staudinger (Willstätter′s successor at the then newly named “Eidgenössische Technische Hochschule” in Zürich) that polymers were covalently bonded molecular chains and even before the existence of macrocycles—the putative components of catenanes—was known.[3] Half a century on, following pioneering studies on the synthesis of medium and large cyclic molecules by the research groups of Ružička,[4] Ziegler,[5] Prelog,[6] Stoll,[7] and others,[8] and the discovery of cyclic molecules in various synthetic polymer-forming reactions,[9] speculation about the existence of catenanes—and their designed synthesis—gained fresh impetus. In 1953 Frisch, Martin, and Mark postulated that the liquid/waxy appearance of high-molecular-weight polysiloxanes might be due to the presence of large interlocked rings acting as plasticizers.[10] The following year Lüttringhaus and Cramer began working on synthetic routes to catenanes (e.g. **1**), building on efforts by Cramer’s group to make rotaxanes as early as 1950.[11] An unsuccessful early attempt to make a catenane, based on the cyclization of an inclusion complex (**2**) of a *para*-disubstituted benzene (**3**) within a cyclodextrin (**4**), was published in 1958[11] (Scheme 1 a).[12]

The first [2]catenane for which evidence was put forward in support of its structure was synthesized two years later by Wasserman.[1] IR spectroscopy indicated that a small (ca. 0.0001 %)[1b fraction of the product obtained through the acyloin condensation of diester **6** in the presence of deuterated macrocycle **5** contained both deuterium atoms and the acyloin (-COCHOH-) group. It seems that this combination of functional groups in a single molecule can only result from this reaction through the formation of a catenane, **7** (Scheme 1 b), although no definitive proof of the structure (e.g. from mass spectrometry) was ever presented.[1b After oxidative cleavage of the acyloin group in the fraction of the reaction products containing the putative catenane, a small amount of **5** was recovered, again supporting the suggestion that the deuterated cycloalkane was a component of a mechanically interlocked molecule. To date, **7** remains the only catenane synthesized that incorporates a fully saturated cycloalkane.

**Scheme 2:**
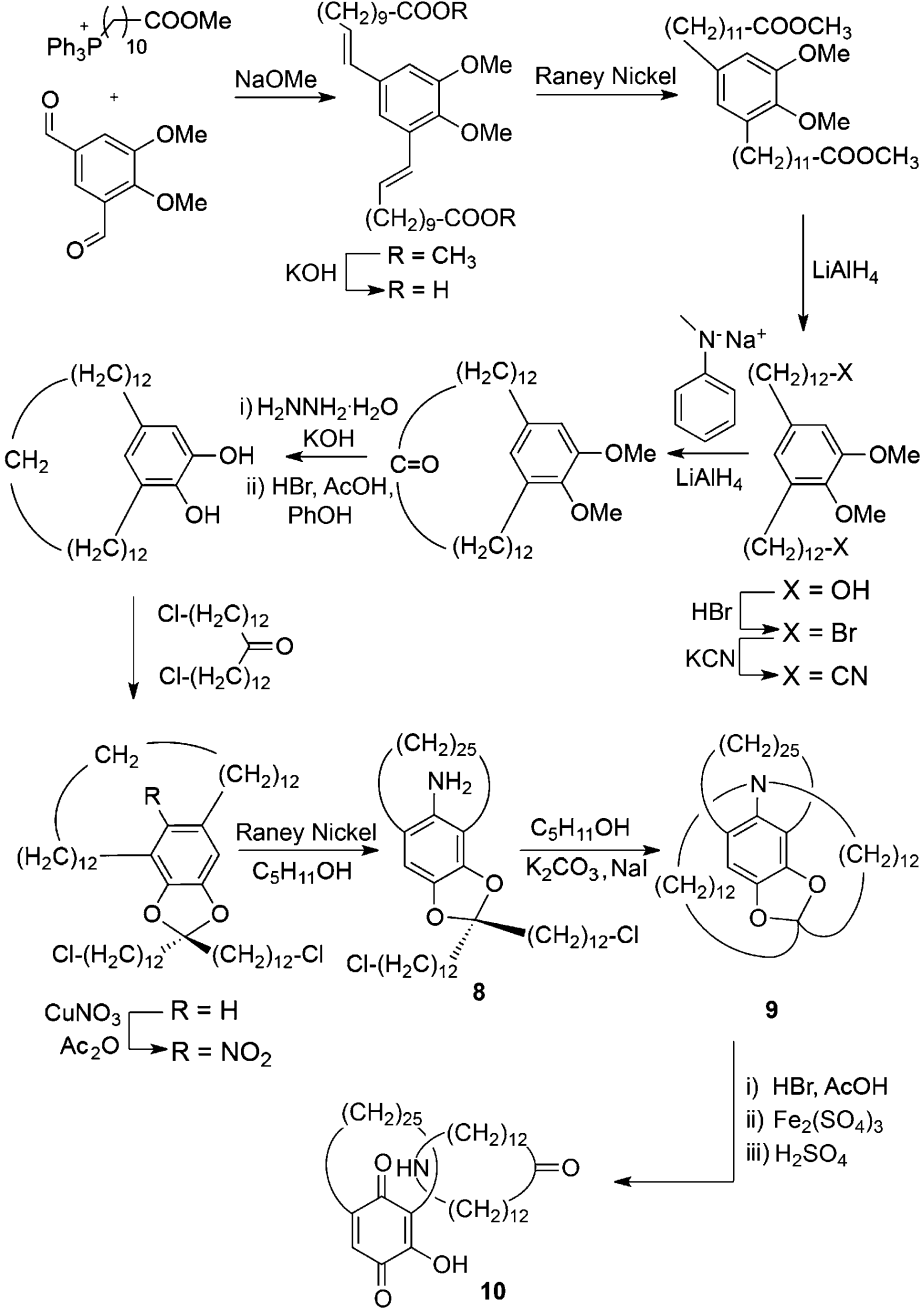
Schill and Lüttringhaus's directed synthesis of a [2]catenane (1964).[14]

In their seminal 1961 discussion of “Chemical Topology”[2] Frisch and Wasserman began to consider ways of overcoming the limitations of statistical methods, and suggested the use of molecular scaffolds and directed synthesis to obtain mechanically interlocked links and knots.[13] The experimental realization of a covalent-bond-directed catenane synthesis by Schill and Lüttringhaus followed shortly afterwards (Scheme 2).[14] In their stepwise route, the positioning of the amino group within the macrocyclic cavity of intermediate **8**, together with the electrophilic alkyl chlorides situated above and below the plane of the macrocycle, is key in directing intramolecular cyclization to give the threaded structure **9**. Cleavage of the aryl–nitrogen bond afforded [2]catenane **10** in 15 steps from a readily obtainable phosphonium salt. This first example remained one of the most efficient demonstrations of catenane synthesis for almost 20 years, only superseded by Sauvage′s application of template methods (see Section 2.1). The ingenuity demonstrated in the Schill and Lüttringhaus 1964 synthesis was the forerunner of chemists applying their imagination and skills to the synthesis of interlocked molecules for the next half-century.

**Scheme 3:**
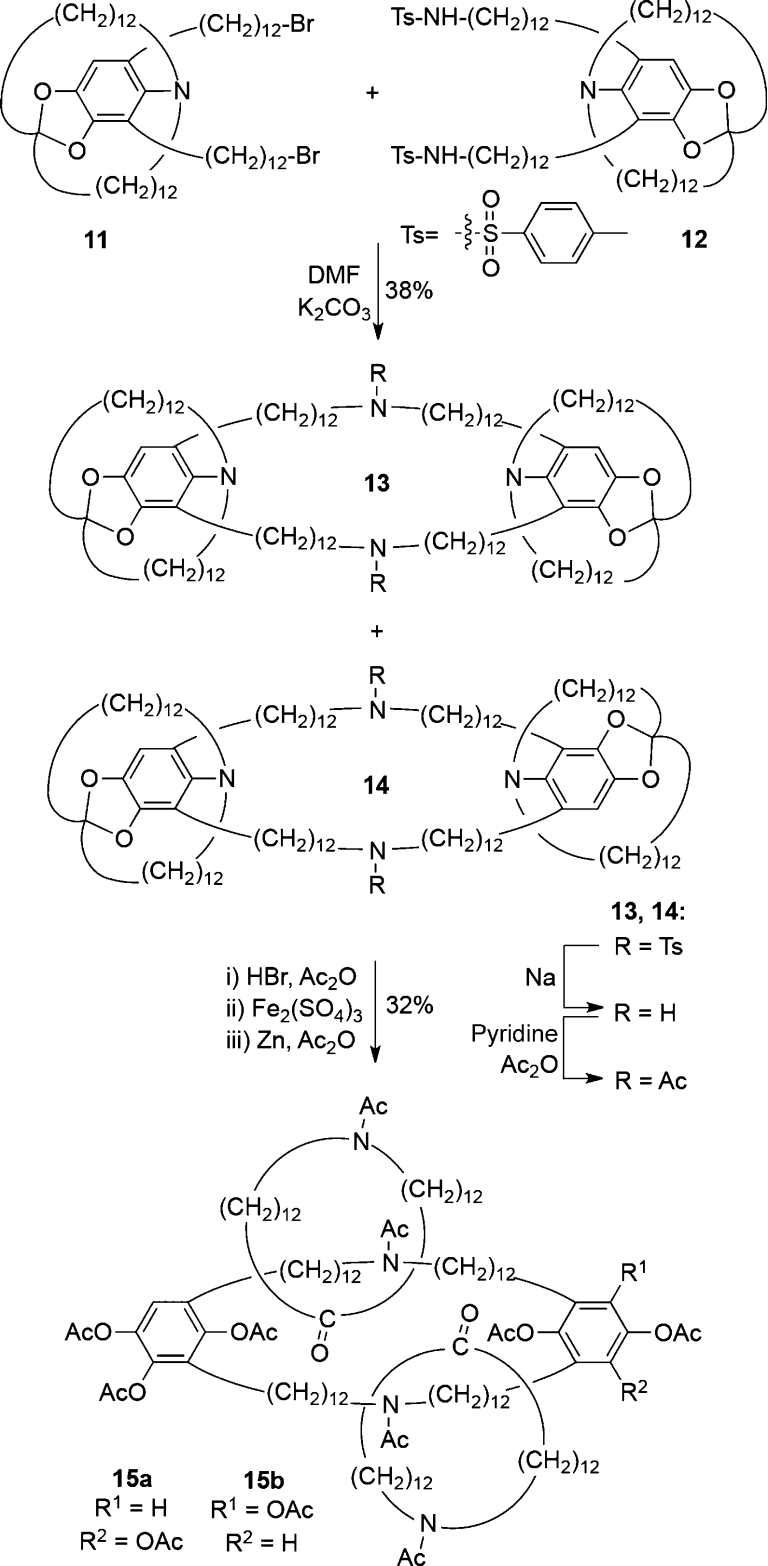
Schill's directed synthesis of a [3]catenane (1969).[15]

In the decade following the synthesis of [2]catenane **10**, Schill et al. extended the “directed synthesis” approach to produce [3]catenanes (e.g. Scheme 3).[15] Macrocyclization of dibromide **11** with diamine **12** gives an inseparable mixture of precatenane isomers **13** and **14**. Cleavage of the directing amino groups afforded a [3]catenane as a mixture of isomers **15 a** and **15 b**. Schill et al. also investigated alternative synthetic routes to circumvent the formation of [3]catenane isomers, but these ultimately proved unsuccessful.[16]

**Figure 1:**
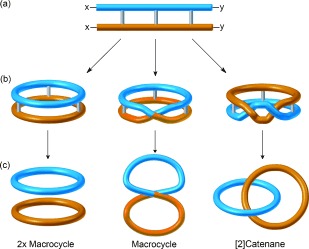
The “Möbius strip” approach to generating different topological isomers. The macrocyclization of a ladder-shaped molecule (a) can form a range of cyclic molecules containing different numbers of twists (b). Cleavage of the rungs yields different topological isomers (c).

In the late 1950s Frisch and Wasserman[2] and van Gulick[17] independently conceived an alternative synthetic strategy to knots, catenanes, and other topologically complex structures: the “Möbius strip approach”. A Möbius strip is a paradromic ring with a half-twist: that is a surface with only one side and one boundary component, formed by twisting a strip and connecting the ends to form a loop. If a paradromic ring is cut along its center, a single ring, link, or knot results (a half-twist Möbius strip affords a ring, even numbers of half twists give links, odd numbers of half twists give knots). In chemical terms the macrocyclization of a ladder-shaped molecule can, in principle, generate loops with differing numbers of twists (Figure 1). Cleavage of the ladder “rungs” would afford various topoisomers (Figure 1 c).

**Scheme 4:**
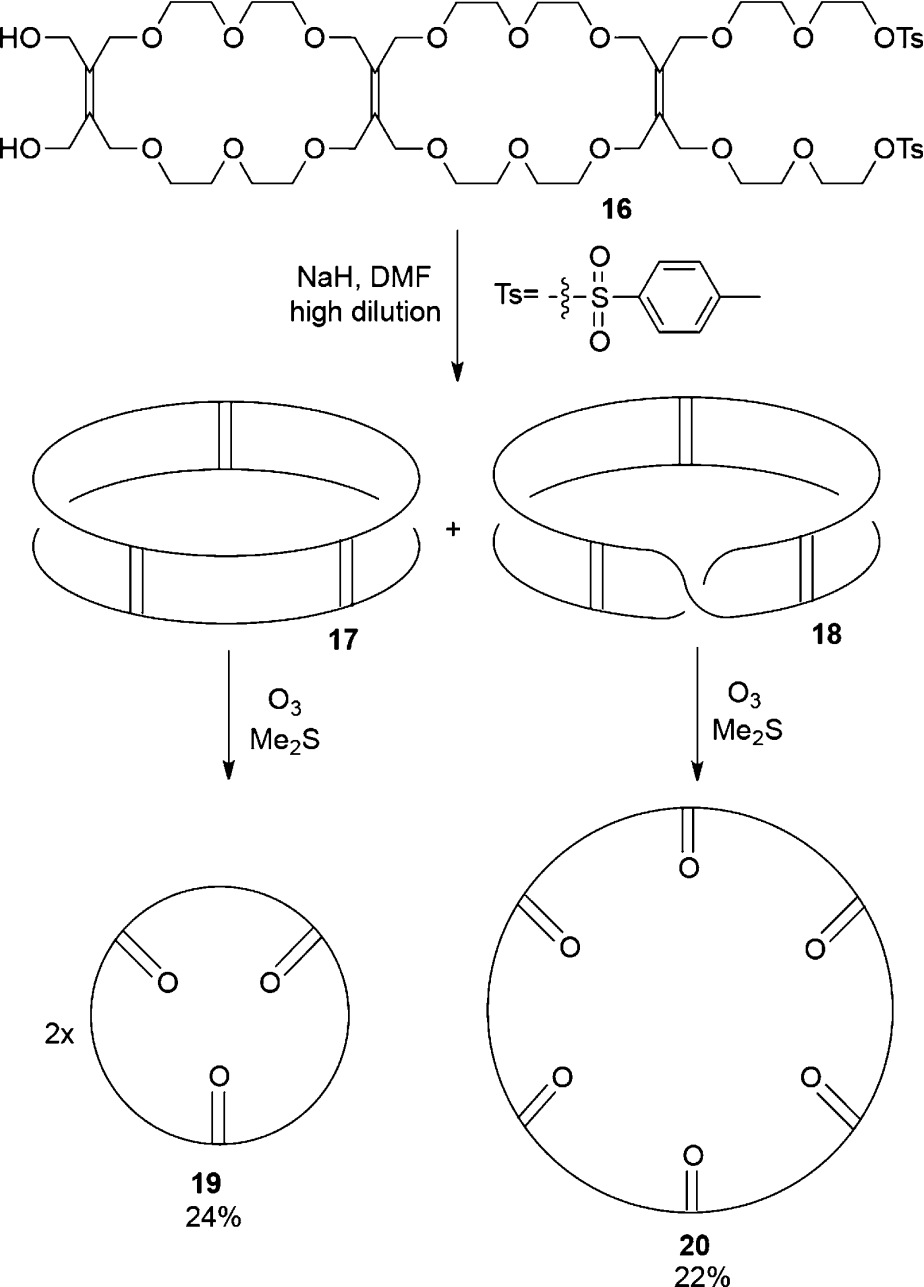
Walba′s synthesis of a molecular Möbius strip.[19]

A “Möbius strip mechanism” was originally proposed to account for the apparent formation of catenanes during the metal-catalyzed olefin metathesis of various cycloalkenes.[18] However, this explanation fell out of favor when the mechanism of olefin metathesis was shown to involve metal-alkylidene and metallacyclobutane intermediates, incompatible with a paradromic ring mechanism for the formation of interlocked molecules.[13a In the early 1980s, Walba et al. synthesized a number of molecular strips based on a tetraether of tetrahydroxymethylethylene (Scheme 4).[19] Cyclization of **16** yielded two products, identified as untwisted cylinder **17** and the half-twist Möbius strip **18**. Scission of the strips was carried out by ozonolysis. Cylinder **17** afforded two molar equivalents of triketone macrocycle **19**, while the half-twist Möbius strip **18** generated the hexaketone macrocycle **20**. However, the double and triple twist paradromic rings, which would be precursors to a [2]catenane and trefoil knot, respectively, could not be isolated, probably because of their very low yields of formation.[19b

Although the early directed and statistical methods to catenanes showed that the synthesis of interlocked molecules was experimentally feasible, the low yields and long multistep routes meant that a radically new approach to their synthesis was necessary for catenanes to become more than academic curiosities. The revolution in catenane synthesis started in 1983 when Strasbourg chemist Jean-Pierre Sauvage recognized that the orthogonal arrangement of bidentate ligands in a tetrahedral Cu^I^ complex could be used to generate the crossing points necessary for catenane formation.[13c Template synthesis has been the basis for forming the vast majority of catenanes ever since (Sections 2–4). However, before discussing the phenomenal advances in catenane assembly achieved during the “template synthesis era”, it is first useful to connect chemical structure with the nomenclature used to describe the topology of links in mathematics (Section 1.2), and to put small-molecule catenanes into a broader context by giving a brief account of catenane macromolecules found in biology or assembled using biopolymer building blocks (Sections 1.3 and 1.4).

### 1.2. Link Topology

In the 19th century, interlocked and entwined rings captured the attention of mathematician Peter Guthrie Tait. Inspired by Lord Kelvin′s proposal that elements were knotted structures in the aether,[20] Tait set about tabulating different types of knots in the hope that it might be related to the periodic table of elements.[21] Although the association between knots and atoms proved to be incorrect, Tait′s work paved the way to the study of knots and the formulation of the mathematics of topology. Modern knot theory also includes the study of links, which are multiple loops (knotted or not) entwined and linked with one another, whereas knots are formally a single entwined loop. As the same definition used to describe a mathematical link can be applied to catenanes, it follows that all links (with the exception of those with zero crossings) expressed in molecular form are catenanes. Clearly an infinite number of interwoven molecular ring patterns are theoretically possible. Nevertheless, only a handful of different catenane topologies have been made to date.

**Figure 2:**
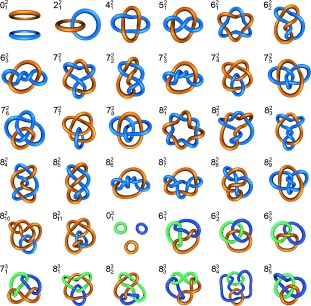
A selection of prime links with up to eight crossing points, and their Alexander–Briggs notation.

**Figure 3:**
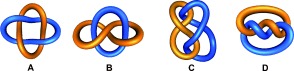
Four different representations of a Solomon link (${4_12 }$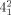).

The Alexander–Briggs notation[22] is commonly used to mathematically describe knots and links. It is written in the form ${{\rm{X}}_zy }$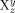, in which *x* corresponds to the number of crossings within the system, *y* is the number of discrete components (loops), and *z* is the order of the knot used to distinguish a given topology from others with the same *x* and *y* descriptors. For example, the simplest type of [2]catenane with two crossings is the ${2_12 }$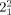 link, also known as a Hopf link (Figure 2). For a given link there can be multiple ways of graphically representing the topology, with interconversion between the different representations achieved through structural deformation and/or translation of the crossing points. For example, Solomon links (${4_12 }$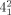) consist of two loops interlocked twice with one another in the simplest depiction of the topology (Figure 3 A), but by deformation, other forms of the Solomon link can be created which are topologically equivalent (Figure 3 B–D).

### 1.3. Naturally Occurring Catenanes in Biopolymers

**Figure 4:**
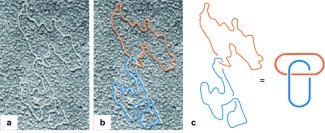
a) Electron micrograph of circular DNA revealing a catenane topology. b,c) Highlighting the two component rings of the DNA catenane as a Hopf link. Modified from Ref. [23] with permission.

Unbeknownst to the chemists devising the early synthetic routes to catenanes, mechanically linked molecular rings occur naturally. In 1967 electron microscopy revealed catenanes made up of circular DNA isolated from the mitochondria of HeLa cells[23] and human leukaemic leucocytes[24] (Figure 4). Since then, many different interlocked DNA topologies have been both discovered and synthesized.[25] Topoisomerase enzymes are responsible for controlling DNA topology.[26] They function by binding to entangled DNA and cutting through the phosphate backbone to introduce gaps in the double helix, which subsequently unwind and disentangle the molecular threads. As enzymes responsible for transcription and replication require access to non-interlocked single strands of DNA to properly read the relevant genetic sequences, the formation and disentanglement of DNA catenanes and knots is of significance for both cell reproduction and gene expression.

**Figure 5:**
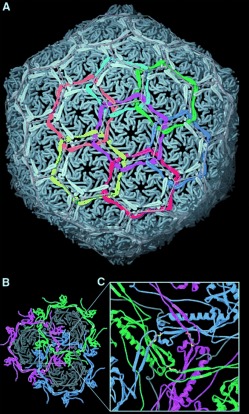
The “chainmail” arrangement of proteins found in bacteriophage HK97s capsid (colored sections highlight the individual protein rings). a) The repeating pattern of interlocking proteins which constitute the spherical capsid. b) A cross-section of the capsid in which three protein rings interlock with one another. c) Magnified view of the position at which protein rings overlap (cross-linking isopeptide bonds are highlighted). Reprinted from Ref. [28] with permission.

Threaded entanglements in proteins, usually in the form of open knots,[27] can be crucial to their tertiary structures and therefore also important for their function. Protein catenanes have also been discovered in natural systems. The X-ray crystal structure of the bacteriophage HK97s capsid[28] shows it to be composed of a network of cyclic proteins interlocked with one another to create a “molecular chainmail” (Figure 5). The relatively thin bacteriophage capsid is thought to gain additional stability by interlocking proteins in this manner, thus providing a means of protecting the genetic information contained within the capsid necessary for virus replication, even in harsh environments. Four other natural protein catenanes are known: 1) *Pyrobaculum aerophilum* citrate synthase (PaCS) proteins,[29] in which the formation of intramolecular disulfide bonds result in an interlocked dimeric assembly of the biopolymers. This feature enhances the proteins thermal stability, which may be particularly important for *Pyrobaculum aerophilum* given that it is a thermophile (i.e. an organism that thrives at high temperatures); 2) two interlocked protein rings in mutant bovine mitochondrial peroxiredoxin III;[30] 3) Recombination R (RecR) proteins used for recombinatorial DNA repair in the *Deinococcus radiodurans* bacterium which form interlocked octamers,[31] an arrangement which it has been suggested may enable the RecR rings to open and close and act as a DNA clamp during the repair process; 4) *E. coli* Class 1a ribonucleotide reductase (RNR) catenanes.[32] Additionally, there is evidence to support the discovery of naturally occurring catenanes in the CS2 hydrolase enzymes found in archaeon *Acidianus A1-3*.[33]

### 1.4. Catenanes Constructed Using Synthetic Biopolymers

**Figure 6:**
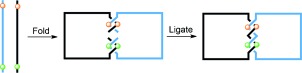
Forming catenanes from single strands of synthetic DNA. Complementary sequences of DNA (represented by colored spheres) fold into helicates, the subsequent ligation of the strands affords catenanes.

In recent years the programmability of DNA recognition sequences and automated synthesis has enabled the preparation of catenanes and other more complicated topological structures from DNA. Synthetic routes to DNA catenanes[34] generally involve the use of single strands of DNA that contain specific sequences of nucleic acids designed to fold into double-stranded helical turns with a complementary sequence contained on a separate thread, thereby creating crossing points between the DNA strands. Catenanes are formed by ligation of the loose ends (Figure 6). An additional level of control can be imparted to the structural assembly by using B or Z DNA to form right- or left-handed helical twists, respectively. By utilizing these approaches Seeman and co-workers have synthesized an array of DNA structures including Borromean rings,[35] a truncated octahedron,[36] and DNA cubes.[37] Recently, efforts have been made to control the relative motion of the rings in synthetic DNA catenanes for their possible use as molecular machines.[38]

Although their design and synthesis presents more challenges than their DNA counterparts, protein catenanes have also been successfully synthesized.[39] The dimeric folding of a tumour suppressor protein p53 to form bisecting concave peptide strands has been exploited such that intramolecular cyclization by native chemical ligation forms a catenane.

## 2. Synthesis of Hopf Link (Singly Interlocked) [2]Catenanes

### 2.1. Catenane Synthesis Promoted by Noncovalent Interactions

The early statistical and directed synthetic approaches to mechanically interlocked molecules[40] suffered from low yields and/or lengthy synthetic procedures (Section 1.1). This meant that catenanes remained firmly in the realm of laboratory curiosities rather than molecular architectures that could be usefully exploited. The situation changed with the application of template synthesis to the preparation of entwined and interlocked structures.[41] This enabled catenanes to be made on a significant scale for the first time. The formation of an interlocked molecule requires the physical overlap of molecular strands to create crossing points prior to covalent capture, a task for which templates are ideal, as they act to gather molecules into preorganized assemblies.[42] In addition to the often high yields and relatively short synthetic pathways associated with template synthesis, predictability and reliability in template preorganization enables the rational design of synthetic pathways to more complex topologies. Over the past three decades these have afforded a number of fascinating (and often beautiful) mechanically interlocked molecular architectures of ever increasing complexity.

#### 2.1.1. Passive Metal Templates

**Scheme 5:**
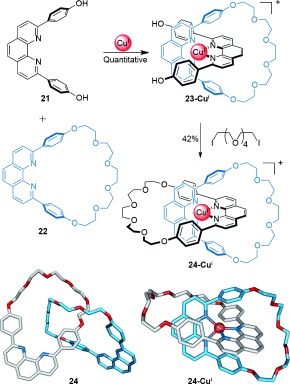
Sauvage's metal-template synthesis of a [2]catenane by Williamson ether macrocyclization of the tetrahedral coordination complex 23-Cu^I^, formed by coordination of ligand 21 and macrocycle 22 to Cu^I^.[43]

The organization of ligands about transition-metal cations has proven to be the one of the most practical, versatile, and reliable methods for creating molecular crossing points,[41], [42b,[c,[e,[f,[h,[i owing to the various well-defined coordination geometries that transition-metal complexes adopt. Sauvage′s group was the first to exploit this in the synthesis of interlocked molecules and introduced metal template [2]catenane synthesis in 1983 (Scheme 5).[43] Their original system employed a hydroxy-functionalized 2,9-diphenyl-1,10-phenanthroline (dpp) ligand **21** and related macrocycle **22** that adopt a threaded structure on coordination to a tetrahedral Cu^I^ ion. The perpendicular ligand arrangement generates a crossing point. Subsequent “clipping” by a Williamson ether macrocyclization reaction covalently captured the [2]catenane **24-Cu^I^** in 42 % yield. The copper ion could be removed quantitatively from catenate **24-Cu^I^** with potassium cyanide to afford the metal-free catenand **24** (Scheme 5).

**Scheme 6:**
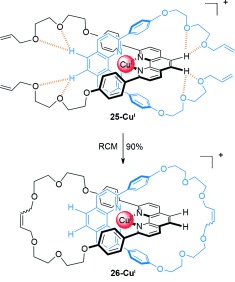
Sauvage's high-yielding olefin metathesis macrocyclization to form [2]catenane 26-Cu^I^.[44] Electrostatic interactions between the glycol ether oxygen atoms and the aromatic protons of the metal-coordinated dpp ligands may help promote intramolecular macrocyclization.

Developments in covalent-bond-forming reactions are often quickly exploited in the synthesis of interlocked molecules. A double ring-closing olefin metathesis (RCM) macrocyclization reaction improved the [2]catenane yield from suitably functionalized dpp ligands[44] to 90 % (Scheme 6).

A significant feature of metal-template catenane synthesis is the possibility of removing the metal ion(s) after covalent capture of the interlocked topology (e.g. catenane **24**, Scheme 5). With the template removed, the macrocycle components are generally able to move with respect to each other with little or no intercomponent noncovalent interactions impeding the motion, within the topological constraints.[45]

Since the original metal-template strategy with dpp ligands and Cu^I^ ions was introduced, catenanes have been synthesized with a range of different metal templates, including the use of octahedral,[46] trigonal bipydramidal,[47] square planar,[48] and linear[49] transition-metal templates. Recently, lanthanide metal ions have also been utilized to template the formation of mechanically interlocked molecules.[50]

**Scheme 7:**
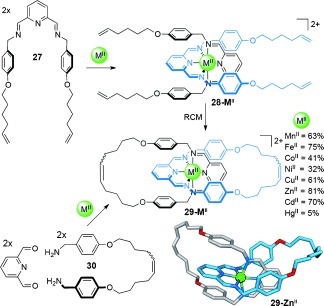
Octahedral [2]catenanes 29-M^II^ formed around a range of divalent metal ions through double RCM reactions from precatenane 28-M^II^ or assembled by imine bond formation.[46d The quoted yields refer to the double RCM route. Stabilizing π–π interactions between the phenyl and the pyridyl rings likely play a significant role in the assembly process.

An additional layer of control can be achieved in metal-template catenane synthesis by using reversible reactions to maximize the yield of the most thermodynamically favorable product, which can often be designed to be the catenane. Imine bond formation is a particularly attractive reaction for preparing metal-template-interlocked molecules; it occurs under mild conditions and its reversible formation allows “error-checking” during the assembly process (Scheme 7).[46d, [51]

Ligand **27** is based on the structure successfully used to assemble hydrogen-bond-assembled benzylic amide catenanes and rotaxanes.[52] It is modified to incorporate a tridentate 2,6-diiminopyridine coordination motif that allows two distinct routes for the preparation of [2]catenanes. One uses precatenane complex **28-M^II^**. Subsequent double macrocyclization by RCM of the terminal alkenes affords the [2]catenane **29-M^II^** in good yields. Alternatively, catenane formation can be carried out under thermodynamic control by exploiting the reversibility of imine bond formation between the bis(benzylamine) chain **30** and 2,6-diformylpyridine in the presence of a divalent metal salt (Scheme 7). The solid-state structures of several of the catenates show interligand aromatic-stacking interactions which are a feature of benzylic amide catenanes and rotaxanes and serve to favor the assembly of the interlocked structure rather than macrocyclic isomers.

**Scheme 8:**
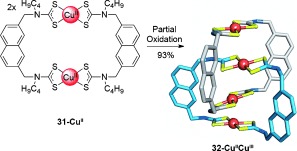
Beer's mixed-valence [2]catenane generated from partial oxidation of macrocycle 31-Cu^II^.[53] Alkyl side chains have been omitted from the crystal structure for clarity.

The use of attractive metal–metal interactions to template the formation of catenanes has been investigated as an alternative to utilizing their coordination geometries. Beer and co-workers found that [2]catenane **32-Cu^II^Cu^III^** (Scheme 8) was assembled through the rearrangement of macrocycle **31-Cu^II^** following its partial oxidation.[53] Charge-transfer interactions between the Cu^II^ and Cu^III^ centers in the mixed-valence catenane probably provide the driving force for interlocking of the macrocycles. This strategy was later extended to the synthesis of mixed-metal Cu^II^-Au^III^ catenanes from the corresponding homometallic macrocycle precursors.[54]

**Scheme 9:**
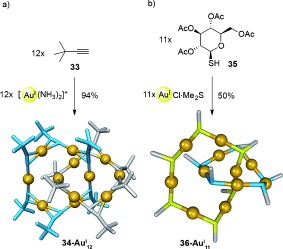
Catenane formation driven by aurophilic interactions from a) Mingos et al. (34-Au^I^_12_)[55] and b) Che et al. (36-Au^I^_11_).[56] In the crystal structure of 36-Au^I^_11_, the sugars have been omitted for clarity, whilst the sulfur atoms that constitute the pentameric ring are shown in blue.

Aurophilic interactions have also been employed to assemble small organometallic fragments into inorganic catenanes. The first such example was reported by Mingos et al. (Scheme 9 a),[55] wherein alkyne **33** was treated with [Au(NH_3_)_2_]^+^ to generate [2]catenane **34-Au^I^**_**12**_. The combination of η^1^-Au-η^1^, η^2^-Au-η^1^, and η^2^-Au-η^2^ alkyne-Au^I^ coordination modes established between the components generates interlocked hexameric macrocycles that are stabilized by the multiple aurophilic interactions between the Au^I^ centers. Au^I^–thiolate interactions have also been employed to generate a [2]catenane (Scheme 9 b).[56]

#### 2.1.2. Active Metal-Template Synthesis

Whilst passive metal templates often provide an effective means of gathering and organizing ligands into interlocked architectures (or precursors to such structures), they do not take advantage of another common feature of transition metals, namely the ability to catalyze covalent-bond-forming reactions. In the last decade, several “active metal-template” strategies to interlocked molecules have been developed.[57] In active template synthesis the metal ion plays a dual role, acting as a template to entwine or thread the building blocks while also actively catalyzing the bond-forming reaction that covalently traps the interlocked structure. In active metal template reactions substoichiometric amounts of metal can often be employed,[58] whilst the use of nonpermanent recognition motifs also allows for the traceless synthesis of interlocked molecules.[59] Initial investigations into the utilization of active metal templates focused on the synthesis of rotaxanes,[58]–[60] but these were later extended to include catenanes[61] and a trefoil knot.[62]

**Scheme 10:**
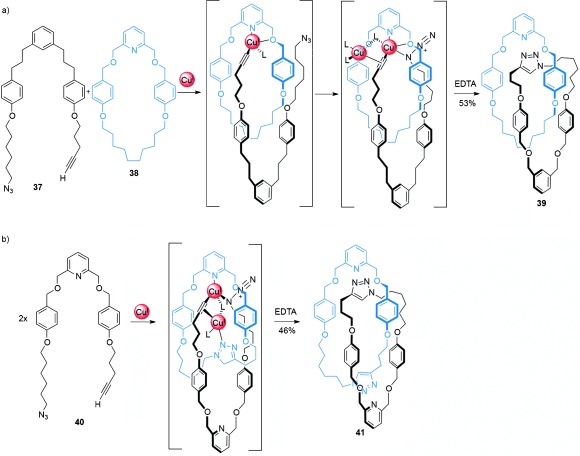
The active metal-template synthesis of a) heterocircuit [2]catenane 39 and b) homocircuit [2]catenane 41 by CuAAC macrocyclization reactions.[61a

A commonly used reaction in active metal-template synthesis is the CuAAC click reaction.[63] The synthesis of a heterocircuit (comprised of two different rings) [2]catenane was achieved by macrocyclization of thread **37** around macrocycle **38**[61a (Scheme 10 a). First a Cu^I^ cation coordinates both to a chelating pyridyl within macrocycle **38** and the complementary azide and alkyne functional groups within **37** to gather the loose thread ends within the cavity of the macrocycle, and thus create a crossing point. Covalent capture of the interlocked product occurs by the Cu^I^-catalyzed cycloaddition reaction between the thread ends to generate [2]catenane **39** (53 % yield). The synthesis of an analogous [2]catenane with a modified macrocycle containing a bipyridine chelating site was also demonstrated, although longer reaction times and higher concentrations were required.

The system was also adapted for a double macrocyclization process using thread **40** (Scheme 10 b), in which a macrocycle is formed in situ by macrocyclization of the thread. A second thread macrocyclization follows through the macrocycles cavity as before, producing a homocircuit (comprised of two identical rings) [2]catenane **41**. The effectiveness of the active metal-template reaction in being able to catalyze two distinct covalent-bond-forming reactions and also act as a template is apparent, as the [2]catenane was formed in reasonable yield (46 %) with little of the non-interlocked macrocycle by-product obtained (<7 %).

In addition to the use of CuAAC click reactions in active metal-template synthesis, [2]catenanes have been prepared using the Cadiot–Chodkiewicz coupling between a terminal alkyne and a bromoalkyne,[61a as well as an oxidative alkyne homocoupling, both Cu^I^-catalyzed reactions.[61b

#### 2.1.3. Catenanes Assembled through π–π Stacking Interactions

**Scheme 11:**
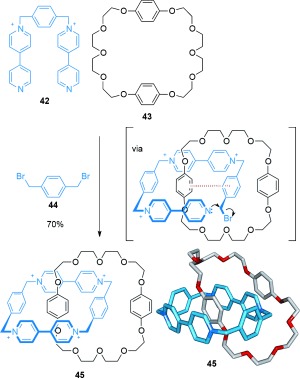
Stoddart's first [2]catenane, featuring π-electron-rich/π-electron-poor aromatic stacking.[65]

Stoddart et al. carried out a seminal research program on the synthesis of catenanes and rotaxanes by exploiting the interactions of π-electron-rich (donor) and π-electron-deficient (acceptor) aromatic rings to direct the threading of molecular strands through macrocycles.[64] The first example was reported in 1989, with the synthesis of a donor/acceptor [2]catenane achieved by combining thread **42** with macrocycle **43** and 1,4-bis(bromomethyl)benzene **44** in acetonitrile[65] (Scheme 11). Given that dication **42** has no significant interaction with the electron-rich macrocycle **43**, the proposed mechanism is that the thread first reacts with 1,4-bis(bromomethyl)benzene to produce a tricationic species containing an electron-deficient bipyridinium motif, which does bind strongly within the cavity of **43** (Scheme 11, intermediate shown in square brackets). The resulting [2]catenane, **45**, is formed in 70 % yield and can also be formed directly from 4,4′-bipyridine, 1,4-bis(bromomethyl)benzene, and macrocycle **43**.[66] A large number of mechanically interlocked molecular systems have subsequently been developed based on this assembly motif.[67]

**Scheme 12:**
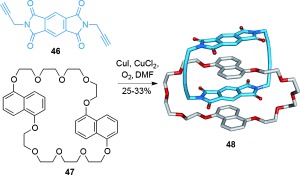
Sanders' use of neutral π-electron-rich and poor motifs to form a [2]catenane by oxidative coupling of alkynes.[68]

Sanders and co-workers have developed neutral donor–acceptor ligand sets that assemble to form catenanes[68] (Scheme 12). These have improved chemical stability and better solubility in organic solvents than Stoddart′s cationic viologen-based macrocycles and catenanes. Combining two molecules of thread **46** with macrocycle **47**, followed by intermolecular oxidative coupling of the terminal alkynes of **46**, afforded [2]catenane **48**.

**Scheme 13:**
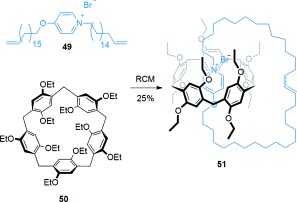
Ogoshi's synthesis of a [2]catenane assembled by threading of a pyridinium salt through the cavity of a pillar[5]arene cyclophane.[69]

Recently, electron-rich pillar[5]arene cyclophanes have been used in the diastereoselective synthesis of a [2]catenane[69] (Scheme 13). Thread **49**, which contains an electron-poor pyridinium unit, and pillar[5]arene **50** assemble to form a pseudorotaxane. Subsequent RCM under high dilution conditions (0.5 mm) generated the [2]catenane **51** (25 %). Rotation of the alkoxybenzene units of the pillar[5]arene can potentially result in eight different diastereoisomers of catenane **51**, but only one is observed (all the alkoxybenzene units have the same orientation relative to one another).[70]

#### 2.1.4. Hydrogen and Halogen-Bond Templates

**Figure 7:**
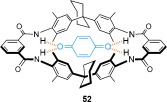
Hunter's benzoquinone receptor.

**Scheme 14:**
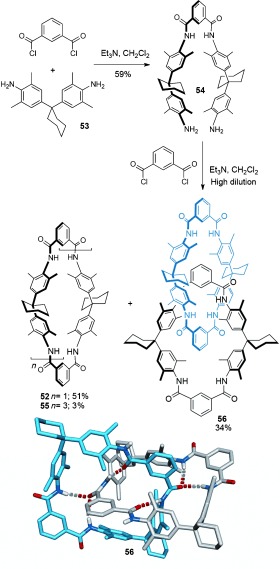
Hunter's synthesis of an amide-based [2]catenane 56 and its X-ray crystal structure (solvent molecules and non-amide hydrogen atoms omitted for clarity).[72], [73] Vögtle reported the synthesis of a very similar [2]catenane shortly afterwards.[74]

In addition to the use of transition-metal templates and π- stacking interactions to direct the assembly of mechanically interlocked molecules, hydrogen bonding has been widely used to promote the synthesis of catenanes. The discovery of the first such system was serendipitous: Hunter and Purvis wanted to investigate **52** as a receptor for *p*-benzoquinone[71] (Figure 7). In an attempt to improve the yield (10 %) of the macrocyclization to obtain **52**, a two-step procedure was carried out in which diamine **54** was isolated prior to the intended macrocyclization (Scheme 14).[72], [73] The reaction of diamine **54** with isophthaloyl dichloride led to the expected macrocycle, **52**, along with two other products which both had twice the molecular mass of the targeted macrocycle. One of the side products was quickly confirmed as the tetrameric macrocycle **55** (Scheme 14). However, structural determination of the third product was more challenging. The molecule′s complex ^1^H NMR spectra and low polarity hinted at the formation of an interlocked structure, which was verified by careful ^1^H NMR analysis as the [2]catenane **56** (Scheme 14). Remarkably, the [2]catenane was obtained in a one-pot double macrocyclization, in which the initial cyclization of **54** with isophthaloyl dichloride affords macrocycle **52** before a second cyclization proceeds through the cavity of the first macrocycle ring, thereby giving **56** in 34 % yield.

Around the same time, Vögtle′s group was investigating the synthesis of similar amide macrocycles[74] and carried out the condensation of diamine **53** and 5-methoxyisophthaloyl dichloride to form a [2]catenane in 10 % yield. A few days after Hunter′s [2]catenane **56** was published, and presumably prompted by Hunter′s detailed ^1^H NMR analysis proving the catenane structure, Vögtle submitted his own paper on the synthesis of a very similar [2]catenane.[75] In both cases the presence of bulky cyclohexyl side groups prevents free rotation of the macrocycles within the resulting [2]catenane.

**Scheme 15:**
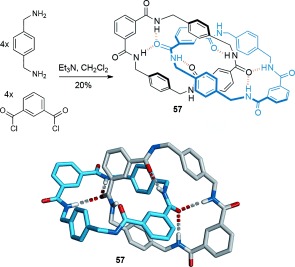
The eight-molecule condensation to form benzylic amide [2]catenane 57.[76]

In terms of dynamic properties and their potential use in molecular machines, the Hunter/Vögtle amide catenanes were largely superseded by the benzylic amide [2]catenane system, serendipitously discovered whilst preparing a macrocyclic receptor for CO_2_.[76] [2]Catenane **57** was prepared in a single step using dibenzylamine and isophthaloyl dichloride (Scheme 15) in a direct eight-molecule condensation reaction that afforded the catenane in 20 % yield. To this day, the one-step, chromatography-free, synthesis of **57** from commercially available chemicals remains one of the simplest ways of accessing a mechanically interlocked molecule. The X-ray structure, the first of an amide catenane, confirmed the amide–amide hydrogen-bonding arrays, as well as showing a network of π–π stacking interactions further stabilizing the structure (Scheme 15). Unlike the previous examples of amide catenanes, the macrocycle components of catenane **57** are able to rotate around one another in solution.[77] Benzylic amide catenanes are generally straightforward to prepare, have high structural versatility as well as variable and controllable ring dynamics.[78]

**Scheme 16:**
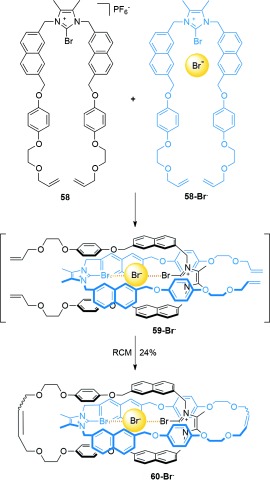
Beer's synthesis of [2]catenane 60-Br^−^ directed by halogen bonding.[80]

The use of halogen bonding[79] in the template synthesis of [2]catenanes has been reported by the Beer group. [2]Catenane **60-Br^−^** was assembled in 24 % yield from RCM of **58** and **58-Br^−^** (Scheme 16).[80] When the macrocyclization was performed in the absence of the bromide ion, using only precursor **58**, no [2]catenane was formed.

**Scheme 17:**
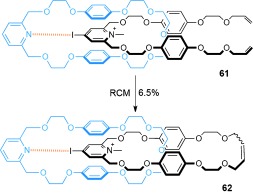
Beer's synthesis of a [2]catenane stabilized by a pyridinium iodide–pyridine interaction.[81]

A pyridinium iodide/pyridine interaction may play a significant role in the assembly of [2]catenane **62** (Scheme 17).[81] The precursor pseudorotaxane complex **61** has a significantly higher association constant in dichloromethane (*K*_a_=180±20 m^−1^) than when the iodine atom is not present (*K*_a_=30 m^−1^). Ring-closing olefin metathesis of **61** generated [2]catenane **62**, albeit in modest yield (6.5 %).

#### 2.1.5. Catenane Synthesis Driven by Hydrophobic Effects

**Scheme 18:**
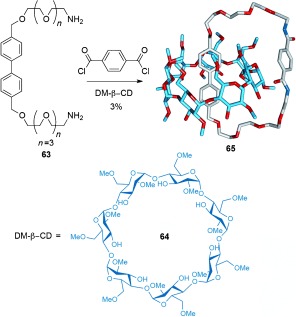
Stoddart's synthesis of [2]catenane 65, promoted by hydrophobic binding.[82]

Solvophobic effects can be used to favor the threading of components to create interlocked molecules (or their precursors), acting to minimize the surface area of hydrophobic motifs that are exposed to a polar solvent by forming inclusion complexes.[82] Although Cramer and Lüttringhaus′s efforts[11] to synthesize a [2]catenane from cyclodextrin complexes in the 1950s were unsuccessful (as outlined in Section 1.1), in the 1990s Stoddart and co-workers were able to isolate an interlocked product by using a similar approach[12] (Scheme 18). The inclusion complex of **63** with heptakis(2,6-di-*O*-methyl)-β-cyclodextrin (DM-β-CD, **64**) was cyclized with terephthaloyl dichloride to afford [2]catenane **65** in 3 % yield.

**Scheme 19:**
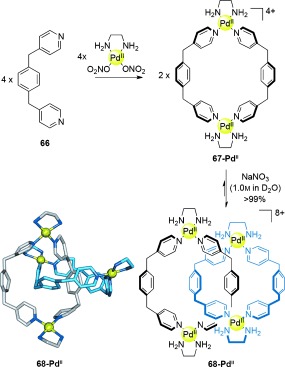
Fujita's “magic ring” [2]catenane synthesis. Reversible coordination of ligand 66 with Pd^II^ generates an interconverting mixture of macrocycle 67-Pd^II^ and [2]catenane 68-Pd^II^, with the [2]catenane energetically favored at high concentrations through hydrophobic binding.[83], [84]

Hydrophobic effects can also be used to generate a thermodynamic bias to favor the interlocking of macrocycles at equilibrium. This concept was discovered serendipitously in the form of Fujita′s “magic rings”[83] (Scheme 19). In basic aqueous solution, macrocycle **67-Pd^II^** spontaneously interlocks to form [2]catenane **68-Pd^II^**. At low concentrations the equilibrium lies towards the macrocycle, however at high concentrations (or at high salt concentrations to increase the hydrophobic effect) the [2]catenane is formed almost quantitatively.[84]

#### 2.1.6. Alkali Metal Cation Templates

**Scheme 20:**
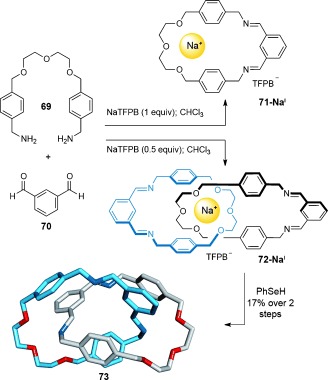
Chiu's Na^I^-template synthesis of a [2]catenane.[86]

Although size-discrimination makes alkali metal cations excellent templates for different-sized crown ethers,[85] their lack of well-defined 3D coordination geometries generally make them unsuitable templates for interlocked molecules. Nevertheless, an example of an alkaline cation template synthesis of a [2]catenane formed under thermodynamic control has been reported by Chiu and co-workers (Scheme 20).[86] The system combines diamine **69** with dialdehyde **70** through reversible imine bond formation in the presence of a sodium salt in a low polarity solvent to give the [2]catenane **72-Na^I^** (Scheme 20). To maximize the template effect, tetrakis[3,5-bis(trifluoromethyl)phenyl]borate (TFPB) was used as a weakly coordinating counterion for the sodium cation. In the presence of 1 equiv of NaTFPB, only macrocycle **71-Na^I^** is observed in the reaction mixture (65 % yield). However, when the reaction is carried out with 0.5 equiv NaTFPB, [2]catenane **72-Na^I^** is formed, which after reduction of the imines, affords [2]catenane **73** in 17 % yield over the two steps.

#### 2.1.7. Anion Templates

**Scheme 21:**
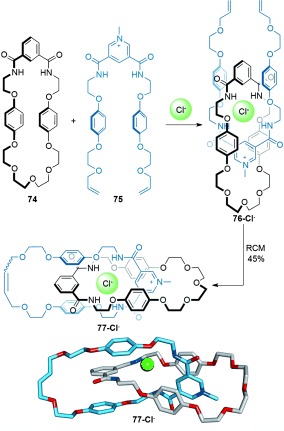
Beer's synthesis of a [2]catenane using a chloride anion template.[88]

The use of anions as templates for interlocked molecules has only developed over the last decade.[87] Whilst some anions (e.g. halides and oxoanions) are capable of forming strong electrostatic interactions due to their small size and high charge density, the absence of predictable coordination geometries in their association complexes often makes it difficult to design the formation of molecular crossing points through their use as templates. Beer and co-workers have overcome some of these problems by utilizing a combination of noncovalent interactions in conjunction with anion templates to impose control over the directionality of the assembly. For example, in the formation of the chloride-complexed [2]catenane precursor **76-Cl^−^** (Scheme 21), macrocycle **74** contains 1) an isophthalamide unit for hydrogen bonding, 2) hydroquinone groups for π–π stacking, and 3) a glycol chain for electrostatic interactions with electron-poor groups and the ability to fold into a low-energy cyclic conformation because of multiple *gauche* effects. Accordingly, [2]catenane **77-Cl^−^** is obtained in 45 % yield (Scheme 21).[88] This strategy was further improved by using two diamidopyridinium-containing threads of **75** assembled around a single chloride anion template followed by double macrocyclization by RCM to afford a [2]catenane in 78 % yield.[89]

#### 2.1.8. Radical–Radical Interactions

**Scheme 22:**
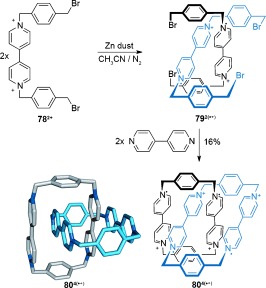
Stoddart's synthesis of a [2]catenane through radical-pairing interactions.[91]

The ability of stable cationic radical viologen species to dimerize in solution has been known for over fifty years,[90] with the pairing of the radicals driven by the resulting closed-shell electron configurations. Stoddart and co-workers recently reported the use of this dimerization phenomenon to assemble [2]catenanes (Scheme 22).[91] Reduction of **78^2+^** to the monoradical species is achieved with an excess of zinc dust, thereby resulting in a threaded dimeric assembly **79^2(.+)^** (Scheme 22). Cyclization with 4,4′-bipyridine affords [2]catenane **80^4(.+)^**. Although the initial radical quenching occurs on exposure to air to give an equilibrium mixture of **80^2.6+^** and **80^.7+^**, the radical species persist for several weeks under ambient conditions, thus requiring forcing oxidation conditions to completely generate the nonradical **80^8+^** species.

### 2.2. Catenanes from Dynamic Combinatorial Libraries

Dynamic combinatorial libraries (DCLs) are systems consisting of molecular building blocks that combine through reversible covalent bonds to produce a mixture (“library”) of different species.[92] As the members of a DCL are in equilibrium, their relative thermodynamic stabilities determine the composition of the library, an equilibrium that can be shifted by changing the reaction conditions (e.g. by adding a substrate that noncovalently binds to, and thus lowers the free energy of, a particular species in the library).

**Scheme 23:**
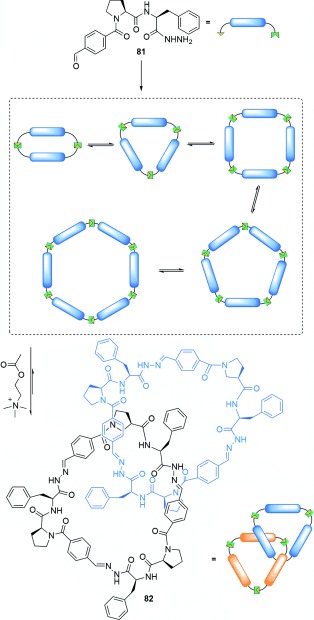
Sanders' [2]catenane formed within a DCL and amplified by acetylcholine biding.[93]

Sanders, Otto, and co-workers discovered a [2]catenane[93] present in a DCL formed by hydrazone exchange of the peptide-based building block **81** (Scheme 23). The building block quickly forms linear oligomers that rearrange over 60 min into cyclic molecules of various sizes. The introduction of acetylcholine to the DCL causes predominant conversion of the library members into **82**, a [2]catenane comprised of trimeric rings. Despite two diastereoisomers being possible (stereoisomers resulting from the directionality of the unsymmetrical rings), only one diastereoisomer is observed. Gagné and co-workers later modified the tripeptide building block to demonstrate the synthesis of a [2]catenane composed of tetrameric rings.[94] In this case, catenane formation is driven by hydrogen bonding, π stacking, and CH–π interactions rather than acetylcholine binding.

**Scheme 24:**
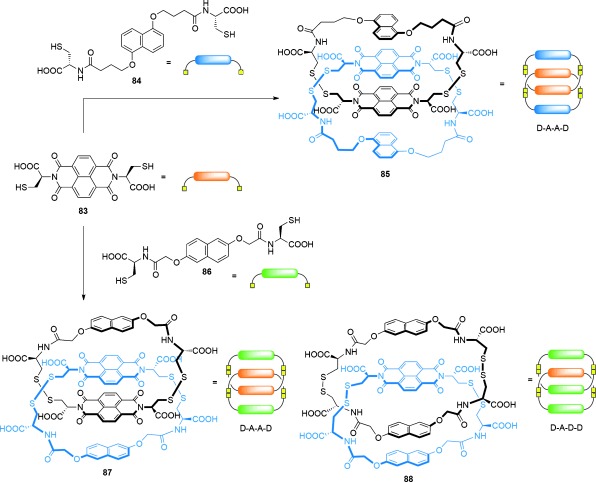
Sanders' partial control over the composition of [2]catenanes formed from a disulfide-based DCL can be achieved by choice of the DN building block. In both cases, the DCLs initially also contained non-interlocked macrocycles. Amplification of [2]catenane 85 was achieved by the addition of either cationic templates to intercalate between the catenanes inner NDI units, or polar salts to enhance hydrophobic effects. Amplification of [2]catenane 87 was achieved by the addition of polar salts, whilst amplification of 88 was achieved by employing a threefold excess of DN 86 relative to NDI 83, and increasing the solvent ionic strength.[95], [96]

[2]Catenanes have also been discovered in DCLs where the components are interchanged through disulfide exchange reactions. Systems developed by Sanders and co-workers made use of components featuring naphthalenediimide (NDI) and dioxynaphthalene (DN) motifs (Scheme 24). Oxidation of dithiol-functionalized NDI **83** and cysteine-functionalized DN **84** in an aqueous solution containing NaNO_3_ produced a [2]catenane **85** composed of heterodimer rings (i.e. containing both an NDI and DN unit) arranged in a donor–acceptor–acceptor–donor (DAAD) fashion.[95] Modification of the DN building block to give **86** afforded the analogous DAAD catenane **87** along with [2]catenane **88** possessing a DADD arrangement of building blocks.[96] The DAAD conformation adopted by [2]catenanes **85** and **87** is such that π–π stacking between the components is maximized, whilst the otherwise unfavorable stacking of π-electron donors is overcome in **88** by hydrophobic forces together with favorable aromatic stacking interactions generated in the DAD stack. The absence of catenanes with the thermodynamically optimal DADA conformation was attributed to the cavity of the acceptor homodimer being too small to permit threading of a donor unit.

## 3. Higher Order Linear and Radial [n]Catenanes

### 3.1. [n]Catenanes

**Figure 8:**
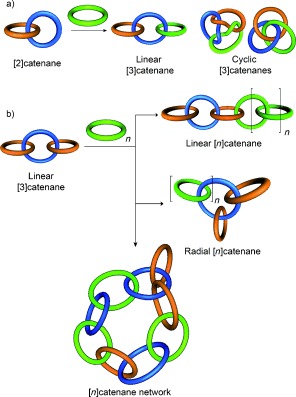
Classification of higher order [*n*]catenanes (*n*>2). a) [3]Catenane topoisomers. b) The addition of macrocycles to a linear [3]catenane generates further topoisomers.

The interlocking of two macrocycles can afford a number of [2]catenane topological isomers (e.g. Hopf link, Solomon link, Star of David catenane etc., see Section 4). The addition of an extra macrocycle allows for an additional type of topoisomerism, in which the connectivity of the linked components varies (e.g. linear and cyclic [3]catenanes, Figure 8 a). The addition of further macrocycles increases both the number and complexity of topological isomers that are possible (Figure 8 b). For example, whilst a [4]catenane can have a linear connectivity of rings, the interlocking of several macrocycles around a single central macrocycle (a “radial” [4]catenane, Figure 8 b) is also possible. The number of discrete topologies possible increases rapidly with the number of components (e.g. [*n*]catenane networks, Figure 8 b).

#### 3.1.1. Linear [n]Catenanes

**Scheme 25:**
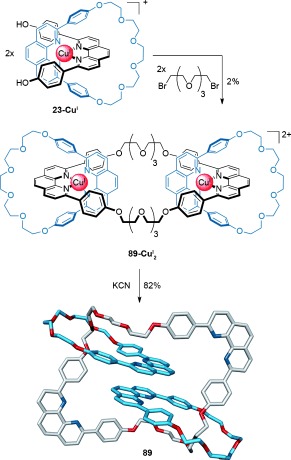
Sauvage's synthesis of a [3]catenane by linking two Cu^I^-complexed pseudorotaxanes.[97], [98]

The first example of a linear [3]catenane was reported by Schill et al., who employed a lengthy multistep synthesis based on his original directed [2]catenane synthesis (discussed in Section 1.1).[15] Later, Sauvage and Weiss employed metal-template synthesis to link two Cu^I^-complexed threaded pseudorotaxanes **23-Cu^I^** to form a [3]catenane **89-Cu^I^**_**2**_, albeit in modest (2 %) yield (Scheme 25).[97] The metal catenate was demetalated with potassium cyanide to give the metal-free [3]catenand **89**.[98] A subsequent development saw the use of the oxidative coupling of terminal acetylenes in place of the Williamson ether synthesis for the intermolecular macrocyclization, thereby increasing the yield of the [3]catenane to 58 %.[99]

**Scheme 26:**
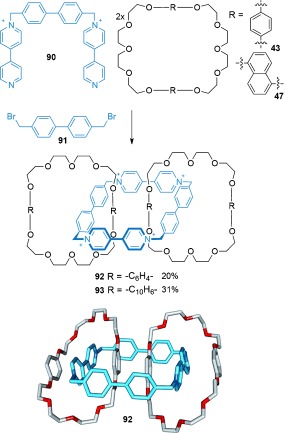
Stoddart's assembly of [3]catenanes.[100]

By increasing the size of the electron-poor macrocycle employed, the Stoddart group were able to promote the linking of two electron-rich macrocycles through a tetracationic cyclophane.[100] The tricationic intermediate resulting from the addition of **90** to **91** is large enough to cyclize around two crown ether macrocycles containing either hydroquinone (**43**) or 1,5-dioxynaphthalene (**47**) rings (Scheme 26). The resulting [3]catenanes **92** and **93** were isolated in 20 % and 31 % yields, respectively.

**Scheme 27:**
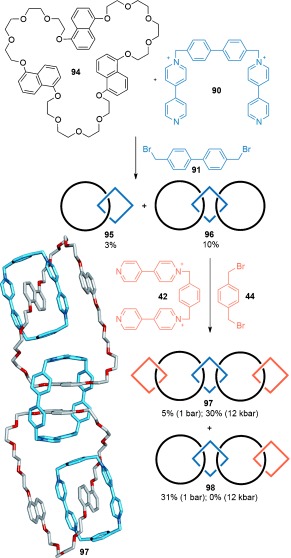
Stoddart's linear [5]catenane “Olympiadane”.[101]

A similar approach was used to assemble a linear [5]catenane the Stoddart group termed “Olympiadane” because of its topology being shared with the symbol of the Olympic games.[101] The additional electron-rich 1,5-dioxynaphthalene groups in the large crown ether macrocycles of [3]catenane **96**, allowed for further cyclization of dication **42** and 1,4-bis(bromomethyl)benzene **44** to afford [5]catenane **97** in 5 % yield and [4]catenane **98** in 31 % yield (Scheme 27). The efficiency of the catenane-forming reaction was improved by the use of ultrahigh pressure conditions (**97**: 30 % at 12 kbar), which also formed the nonlinear [6]- and [7]catenanes in 28 % and 26 % yields, respectively. The rationale for the improved catenane yields using ultrahigh pressure conditions is that the number of components decreases in going from reactants to products.

Although examples of discrete linear [4]- or [5]catenanes are still rare in the literature, other successful strategies for the synthesis of linear [3]catenanes include an amide-based [3]catenane rotary motor (see section 5.2),[102] Fujita′s Pd^II^ [3]catenane,[103] Loeb′s host–guest [3]catenane,[104] and Sanders’ [3]catenane generated from a DCL.[105]

#### 3.1.2. Radial [n]Catenanes: n-1 Macrocycles Threaded onto a Central Macrocycle

**Scheme 28:**
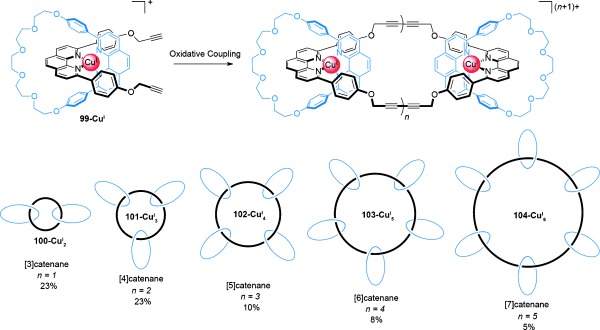
Sauvage's radial [*n*]catenanes generated by the oxidative homocoupling of alkyne-functionalized pseudorotaxanes, detected by ESI-MS. The yields are approximate and are based on *m*/*z* signal intensities from electrospray mass spectrometry. Catenanes 100-Cu^I^_2_, 101-Cu^I^_3_, and 102-Cu^I^_4_ could be isolated by chromatography, structures 103-Cu^I^_5_ and 104-Cu^I^_6_ could not.[106]

Sauvage and co-workers reported the first examples of radial [*n*]catenanes, which they referred to as “multicatenanes”, in 1991.[106] By utilizing the intermolecular cyclization of Cu^I^-complexed pseudorotaxanes as previously described for the synthesis of linear [3]catenanes (Scheme 25), alkyne-functionalized pseudorotaxane **99-Cu^I^** was submitted to oxidative alkyne homocoupling conditions (Scheme 28). Along with the expected [3]catenate **100-Cu^I^**_**2**_, which was formed in 23 % yield, a trimetallic complex was also generated in 23 % yield, tentatively assigned as a [4]catenate consisting of a central 66-membered hexayne ring with three peripheral 30-membered rings. Higher order homologues of these catenates also appeared to be formed in the reaction, with electrospray mass spectrometry of the crude reaction mixture providing evidence of radial [*n*]catenates with up to six rings around a central macrocycle.

**Scheme 29:**
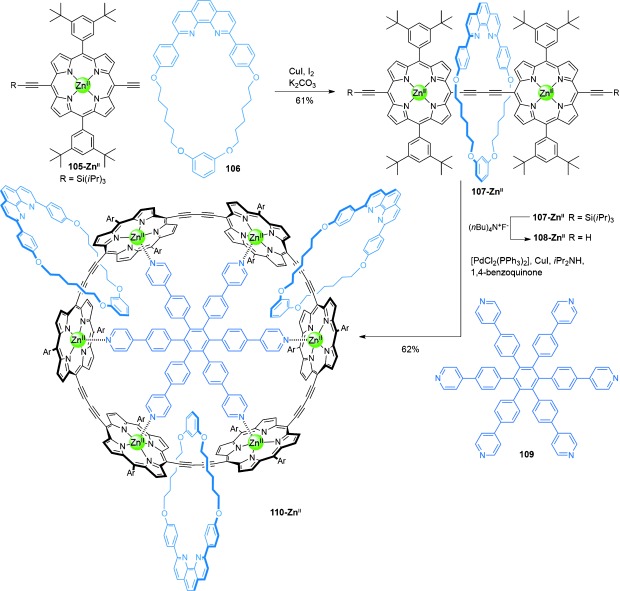
Anderson's synthesis of the radial [4]catenane 110-Zn^II^.[61c

A radial [4]catenane **110-Zn^II^**, comprised of a six-porphyrin nanoring with three mechanically interlocked phenanthroline macrocycles **106**, was assembled by Anderson and co-workers[61c (Scheme 29). The rotaxane porphyrin dimer **107-Zn^II^** was prepared in 61 % yield through an active metal-template Glaser coupling between a monosilyl-protected alkyne-functionalized porphyrin **105-Zn^II^** and a phenanthroline macrocycle **106**. Unmasking of the rotaxane alkyne afforded **108-Zn^II^**. Reaction of rotaxane **108-Zn^II^** under oxidative homocoupling conditions in the presence of hexapyridyl template **109**, gave [4]catenane **110-Zn^II^** in 62 % yield. The template, **109**, could be removed in 89 % yield by treatment with DABCO.

**Scheme 30:**
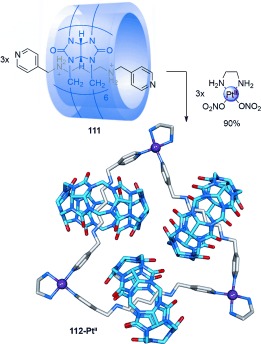
Kim's synthesis of a radial [4]catenane “molecular necklace” 112-Pt^II^, assembled from threaded cucurbituril macrocycles and Pt^II^ connecting units.[108]

**Scheme 31:**
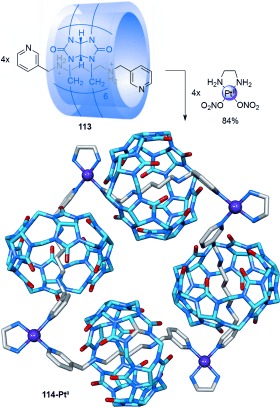
Kim's radial [5]catenane.[109]

Kim connected three cucurbituril-diammonium pseudorotaxanes[107] into a cyclic array through platinum–pyridine coordination to form a “molecular necklace” radial [4]catenane in 90 % yield (Scheme 30).[108] Altering the substitution pattern of the pyridyl group from 4- to 3-substitution gave a radial [5]catenane in 84 % yield (Scheme 31).[109] A thread containing a phenanthroline ligand was used to connect two pseudo[3]rotaxanes with Cu^II^ ions to form a related radial [5]catenane.[110]

**Scheme 32:**
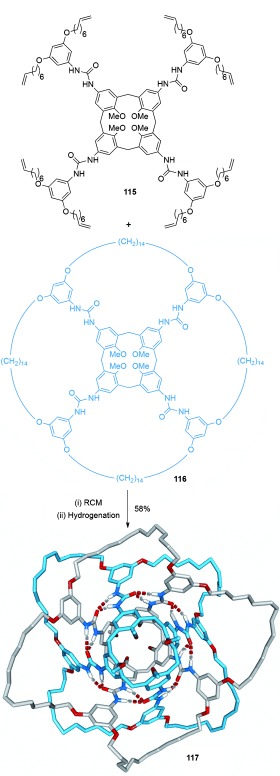
Böhmer's [8]catenane-like structure based on interlocked calix[4]arene dimers. Hydrogen bonding between the urea motifs is illustrated with dashed lines.[111]

Böhmer and co-workers demonstrated the formation of an [8]catenane-like structure by ring closure of the hydrogen-bonded calix[4]arene dimer formed between **115** and **116** (Scheme 32).[111] The synthesis of a related bis([3]catenane) was demonstrated on replacing **115** with a calix[4]arene in which intramolecular cyclization generates two annular cycles.

#### 3.1.3. Other [3]Catenane Topologies

**Scheme 33:**
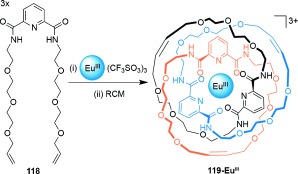
Gunnlaugsson's lanthanide-template synthesis of an apparent ${6_33 }$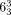 link.[50a

Gunnlaugsson and co-workers have carried out a lanthanide-template synthesis of an interlocked structure, tentatively assigned as a cyclic [3]catenane (Scheme 33).[50a Three equivalents of pyridyldiamide ligands **118** coordinate to a single Eu^III^ cation to generate an assembly from which a [3]catenane can be produced following triple intracomponent cyclization through RCM. However, alternative topologies (macrocycles, [2]catenanes, or knots)[50b may also be produced from the clipping procedure in the absence of sufficient ligand preorganization to direct the ring-closing reactions. Both ^1^H NMR spectroscopy and mass spectrometry indicated that full closure of the complex had occurred following the reaction, and the detection of [2]catenanes containing two equivalents of cyclized **118** as a side product provides evidence for the formation of **119-Eu^III^**, thus indicating a preference for intraligand cyclization within the lanthanide-template assembly.

#### 3.1.4. [n]Catenane Networks

Nature abhors a vacuum and the interweaving and interlocking of rings upon crystallization to form infinitely extended catenane structures is widespread, including examples derived from macrocycles and cages that contain dynamic (e.g. coordination) bonds.[112] As interlocking arises as a means of maximizing van der Waals interactions, and not because of interactions between specific recognition motifs, solvation of the network removes the driving force for the formation of polycatenanes and usually triggers their disassembly. As the consequences of mechanical bonding, such as large amplitude motion of the components, are generally not apparent in close-packed crystalline structures, the utility of such architectures is currently unclear.

**Scheme 34:**
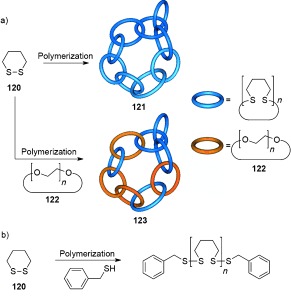
a) Endo's polycatenane networks obtained from polymerization of 1,2-dithiane. b) Synthesis of linear poly(1,2-dithiane).[114]

Some of the earliest suggestions for physical manifestations of mechanical bonding are attributed to polycatenane networks. The anomalous physical properties of polysiloxanes were attributed to the presence of interlocked cyclic polymers in the 1950s[10] as was the depolymerization behavior and rubberlike elasticity of polymeric phosphonitrile chloride.[113] Half a century later Endo et al. proposed[114] a polycatenane network (**121**) is formed from the bulk polymerization of 1,2-dithiane (**120**; Scheme 34 a). Significant differences in the thermal and physical properties of **121** in comparison to linear poly(1,2-dithiane) prepared with benzylmercaptan end groups (Scheme 30 b), and cyclic poly(oxoethylene) **122** infer the formation of a new network topology. Following polymerization of **120** in the presence of **122**, the inability to remove **122** from the resulting polymer mixture, despite the two polymers having significantly different solubilities in methanol, suggests the possible formation of an interlocked network. An analogous investigation into the ring-opening polymerization of 1,4-dihydro-2,3-benzodithiine afforded a polymer with similar properties to **121**.[115]

### 3.2. Interlocked Cage Molecules

**Scheme 35:**
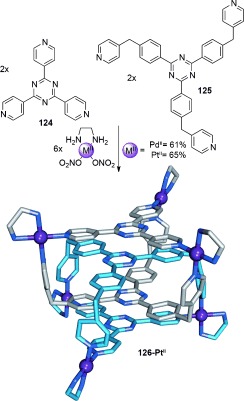
Fujita's triply interlocked cage complex formed by the self-assembly of four organic ligands with six square-planar-coordinating metal cations (Pd^II^ or Pt^II^).[116] The interplanar distance between the aromatic faces of the cages is ideal for generating strong π–π stacking interactions with a separate intercalated cage.

The interlocking of cage molecules can lead to novel catenane topologies through the interpenetration of multiple faces of the cage. The first example of a cage-based catenane was described by Fujita et al. in a one-pot ten-component self-assembly reaction (Scheme 35).[116] In D_2_O, two equivalents of the two tripodal monodentate pyridyl-based ligands **124** and **125** combined with six equivalents of square-planar Pd^II^ or Pt^II^ cations to generate the interlocked cage **126-M^II^** (Scheme 35). Hydrophobic effects, along with intercomponent π–π stacking, drive the formation of the catenane under thermodynamic control.

Several interlocked cage topologies which utilize metal–ligand coordination have since been reported, such as the triply interlocked chiral cages based on Co^II^ and Zn^II^ coordination by Hardie and co-workers,[117] quadruply stranded Pd^II^ cages by Kuroda and co-workers,[118] and interlocked Pd^II^ cages containing phenothiazine by Clever and co-workers.[119]

**Scheme 36:**
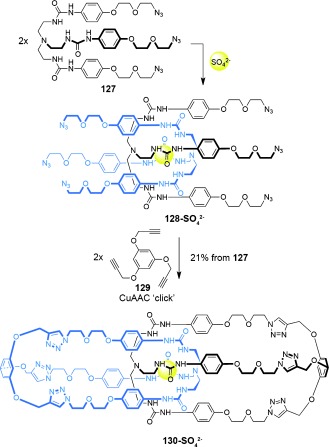
Beer's sulfate anion template synthesis of a catenane cage molecule.[120]

The synthetic routes to interlocked cages are not, however, limited to systems with metal coordination bonds. Beer and co-workers employed a sulfate anion to template the formation of catenane cage complex **130-SO_4_**^**2−**^ (Scheme 36).[120] The product topology was inferred by mass spectrometry, ^1^H NMR spectroscopy, and diffusion ordered NMR spectroscopy (DOSY) experiments.

**Scheme 37:**
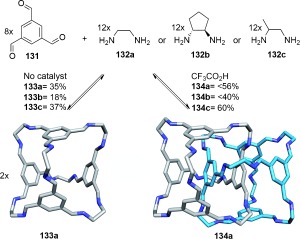
Cooper's formation of discrete interlocked cages mediated by reversible imine bond formation.[121] Non-interlocked cage monomers 133 a–c are formed in the absence of CF_3_CO_2_H.[122]

An alternative strategy to triply interlocked organic cages was serendipitously discovered by Cooper and co-workers.[121] In the presence of CF_3_CO_2_H, tri-aldehyde **131** and a suitable diamine (**132 a**–**c**) affords crystals of cage [2]catenanes **134 a**, **134 b**, or **134 c** (from diamines **132 a**, **132 b**, or **132 c** respectively; Scheme 37). Remarkably, formation of the interlocked product is achieved despite the absence of any apparent recognition motifs to associate the two cage components. It is likely that the catenane preferentially crystallizes from the reaction mixture (the interlocked structure presumably fills the void space more effectively and maximizes van der Waals interactions in the solid state), while the reversibility of imine bond formation produces more catenane in solution through Le Châtelier′s principle during the several weeks of the crystallization process. The resulting catenane cages are, however, relatively stable when taken up in solution under neutral conditions, kinetically trapped by the relatively slow dynamics of imine bond formation.

**Scheme 38:**
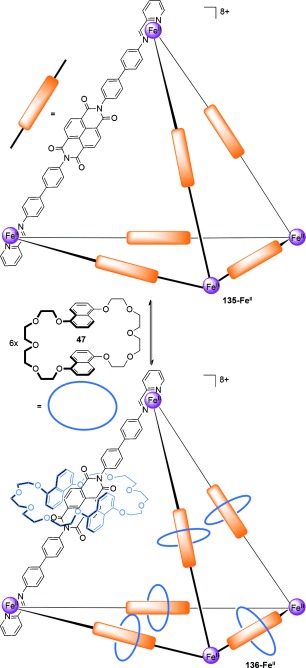
Nitschke and Sanders' [7]catenane based on the dynamic threading of macrocycles around each of the cage vertices.[124]

A [2]catenane cage was recently described by Mastalerz and co-workers,[123] in which reversible boronic ester formation was utilized in a 40-component assembly process to generate a quadruply interlocked catenane. The groups of Nitschke and Sanders have reported the synthesis of a [7]catenane **136-Fe^II^** (Scheme 38) based on a tetrahedral-metal/organic cage complex with six macrocycles threaded around each of the cage vertices.[124]

## 4. Higher Order Entwined [n]Catenanes

The introduction of two additional crossings to a Hopf link (${2_12 }$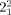 link) generates an inherently chiral doubly interlocked [2]catenane known colloquially as a “Solomon knot”. However, as this topology is not a knot but a link (a ${4_12 }$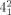 link in Alexander–Briggs notation), it is termed a “Solomon link” by chemists. Adding a further two crossings can generate a triply interlocked or “Star of David” [2]catenane (${6_12 }$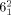 link). Catenanes in which the component rings are entwined about each other multiple times represent a significant challenge to synthetic chemists, with few examples of their successful synthesis reported to date.

### 4.1. Solomon Links

**Scheme 39:**
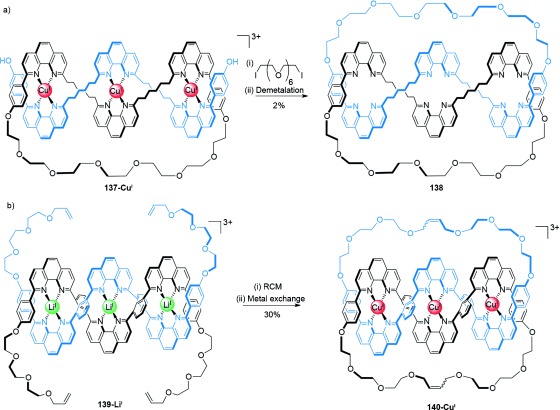
Sauvage's use of trimetallic double-stranded linear helicates to generate a Solomon link in a) a single Williamson ether macrocyclization[125] and b) double macrocyclization by RCM.[126]

The Sauvage group carried out the first syntheses of Solomon links, through the macrocyclization of trimetallic linear double-stranded helicates (Scheme 39).[125] The cyclization of the entwined and threaded complex **137-Cu^I^** through Williamson ether synthesis generated Solomon link **138** in 2 % overall yield after a demetalation step (Scheme 39 a). A more efficient synthesis involved the lithium helicate **139-Li^I^** which was cyclized by double ring-closing olefin metathesis to give the corresponding Solomon link **140-Cu^I^** in 30 % yield after metal exchange (Scheme 39 b).[126]

**Scheme 40:**
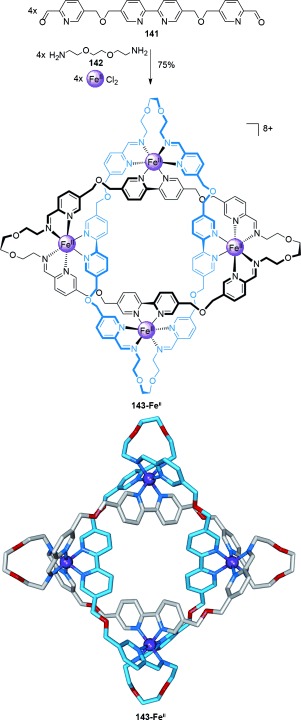
The one-pot synthesis of a Solomon link based on a tetrameric circular helicate.[129]

Although longer linear helicates could theoretically be used to increase the number of crossings, higher order molecular knots and links have yet to be accessed by this strategy. The problem is that as the linear helicate gets longer, the distance between the ends that have to be connected increases and rapidly becomes synthetically impractical.[127] Circular helicates offer the advantage of shorter distances for cyclization, at the expense of a greater number of connections that must take place. Circular helicates have been successfully used to make higher order knots and links, including a Solomon link (Scheme 40).

Bisaldehyde **141** reacts with amines to form tris(bidentate) ligand strands reminiscent of the tris(bipyridine) ligands found by Lehn and co-workers to form tetrameric circular helicates with transition-metal ions.[128] Solomon link **143-Fe^II^** is formed in 75 % yield by heating an equimolar mixture of dialdehyde **141**, diamine **142**, and FeCl_2_ (Scheme 40).[129] The in situ generation of the tris(bidentate) ligands allows for the self-assembly of a tetrameric circular helicate on coordination to Fe^II^, within which parallel ligand strands are covalently connected through the newly formed diimine linkages. The effectiveness of the approach is attributed to the combination of dynamic and reversible imine bonds and metal–ligand coordination correcting any “mistakes” in connectivity, and *gauche* effects within the glycol linkers allowing the low energy turns necessary to cyclize the structure.

**Scheme 41:**
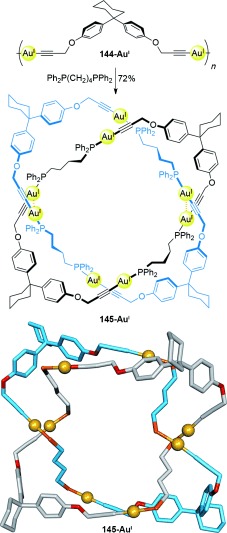
Puddephatt's Solomon link generated from the rearrangement of a dialkyne–Au^I^ polymer on introduction of coordinating diphosphine ligands. Phosphophenyl groups are omitted from the crystal structure for clarity. Au-Au distances; 3.130(2) and 3.239(2) Å.[130]

As with the Hopf link [2]catenanes, Solomon links have been synthesized which contain metal cations as an integral part of the molecular backbone. Puddephatt and co-workers synthesized Solomon link **145-Au^I^** from dialkynylgold polymer **144-Au^I^** on introduction of diphosphine ligands to compete for metal coordination (Scheme 41).[130] Aurophilic interactions between the gold cations in each component ring of the catenane drive the formation of the Solomon link **145-Au^I^**.

**Scheme 42:**
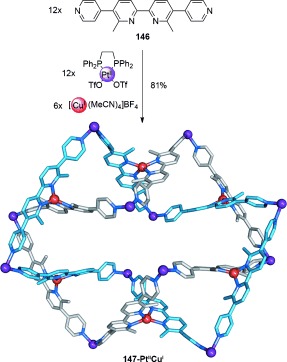
Severin's Solomon link containing a 2:1 mixture of square-planar and tetrahedral coordinated metal cations. Dppp ligands on the Pt^II^ metal centers are omitted from the crystal structure for clarity.[131]

Severin and co-workers recently reported the formation of a large Solomon link using Cu^I^ to template the assembly of pyridine-derivatized bipyridine ligands connected by Pt^II^ coordination (Scheme 42).[131] When ligand **146** was combined with [Pt(dppp)(OTf)_2_] (dppp=1,3-bis(diphenylphosphino)propane) and [Cu(MeCN)_4_]BF_4_ in a 2:2:1 stoichiometry, Solomon link **147-Pt^II^Cu^I^** is formed. Twelve Pt^II^ connectors generate two hexanuclear bowed macrocycles, while six Cu^I^–bipyridine complexes generate the interwoven topology. Other examples of Solomon links include systems closely related to Stoddart′s Borromean rings,[132] and a recent report suggesting a Solomon link is formed in a dynamic combinatorial library.[133] A related “Solomon cube” topology has been reported by Hardie and co-workers.[134]

### 4.2. A Star of David [2]Catenane

**Scheme 43:**
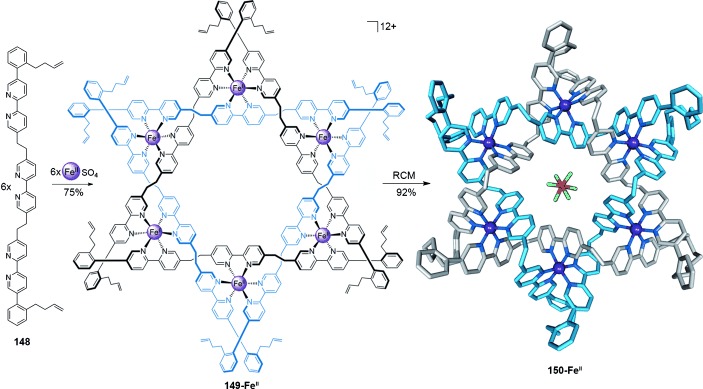
A Star of David [2]catenane synthesized by RCM of a hexameric circular helicate scaffold. A PF_6_^−^ ion occupies the central cavity of the helicate in the X-ray crystal structure of 150-Fe^II^, oriented to direct the fluorine atoms towards the twelve electron-poor protons that line the walls of the cavity (CH⋅⋅⋅F distances 1.88–2.43 Å).[135]

In its simplest representation, the Star of David topology (${6_12 }$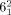 link) consists of two rings entwined about each other three times, thereby creating six alternating crossings. In molecular terms it can be considered a triply interlocked [2]catenane. Like the Solomon link (${4_12 }$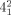 link), the topology is intrinsically chiral. The synthesis of a Star of David [2]catenane was reported by connecting the end groups of a hexameric circular helicate by RCM (Scheme 43).[135] The X-ray crystal structure confirms the structure and topology. Key to the synthesis is the use of the *ortho*-substituted phenyl group in the alkene linker. The twist of the phenyl group restricts the conformations of the linker directing formation of the correct connectivity. Demetalation of **150-Fe^II^** afforded the wholly organic Star of David [2]catenane.

### 4.3. Borromean Rings

Molecular Borromean rings, the simplest type of Brunnian link, are [3]catenane topoisomers in which none of the component rings are linked, but also cannot be separated without breaking one of the rings.[136]

**Scheme 44:**
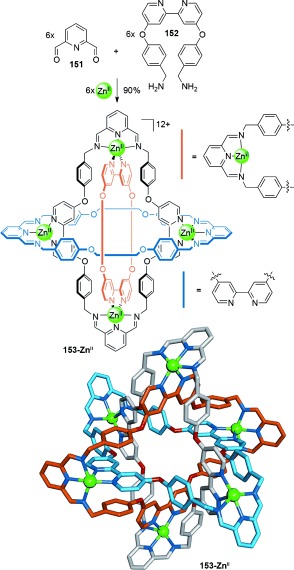
Stoddart's Borromean rings, with the carbon atoms in each ring of the interlocked topology highlighted in different colors (light blue, orange, and gray). Additional anions occupying the sixth coordination site in the Zn^II^ cations have been omitted for clarity. The Zn^II^ centers are all crystallographically equivalent, with distorted octahedral geometries (*cis*-N-Zn^II^-N bond angles 72.5(2)–109.5(3)8). Extensive π stacking occurs between the phenyl rings and bipyridine groups (distances: 3.51 and 3.72 Å).[137]

The Stoddart group synthesized molecular Borromean rings **153-Zn^II^** through the one-pot reaction of six dialdehydes (**151**), six diamines (**152**), and six Zn^II^ ions (Scheme 44).[137] The Borromean ring topology was confirmed by X-ray crystallography, which also revealed π-stacking interactions between the phenoxy and bipyridyl rings that likely aid the assembly process. The Zn^II^ templates could be removed from the structure by reduction of the imine bonds with NaBH_4_, followed by treatment with ethylenediaminetetraacetic acid (EDTA) to afford the demetalated Borromean rings.[138] The Borromean rings were shown to self-assemble from the same ligand set with other metal templates[139] such as Cu^II^, Co^II^, Mn^II^, and Cd^II^, whilst employing equimolar amounts of Zn(OAc)_2_ and Cu(OAc)_2_ in an attempt to form heterometallic Borromean rings resulted instead in a heterometallic Solomon link.[132a

**Scheme 45:**
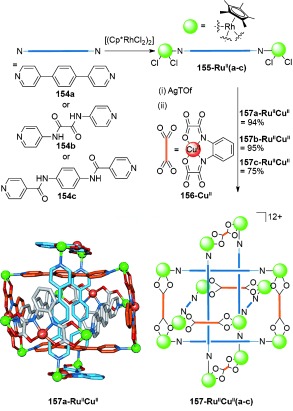
Jin's Borromean rings comprised of RhCp* metallarectangles. The Cp* ligands on the Rh metal centers are omitted from the crystal structure for clarity.[140]

Molecular Borromean rings were also discovered by chance by Jin and co-workers during their investigations of the synthesis of Cp*Rh (Cp*=η^5^-C_5_Me_5_) metallarectangles (Scheme 45).[140] Ligands **154 a**, **154 b**, or **154 c** were coordinated to [(RuCp*Cl_2_)_2_]. Chloride abstraction with AgOTf, followed by addition of **156-Cu^II^** affords Borromean rings **157-Ru^II^Cu^II^(a–c)**. The relative lengths of the ligands was found to be crucial to the formation of Borromean rings; too long or too short, or with a non-optimal ratio of lengths, gave non-interlocked metallarectangles instead.

## 5. Catenanes as Switches, Rotary Motors, and Sensors

The interlocked architecture of catenanes can be exploited for function in several different ways. The dynamics of the large amplitude motions that their components can undergo make catenanes attractive candidates for utilization in molecular machines.[141] The change of the relative positioning of the rings can be used as a switch, or the net directionality of 360° rotation of one ring with respect to each other can be used as the basis for a rotary motor.[142] The cavity formed by interlocked rings can be used to hold functional groups in precise positions in 3D space, a property that can be used to bind substrates with exquisite specificity.

### 5.1. Catenane Switches

**Scheme 46:**
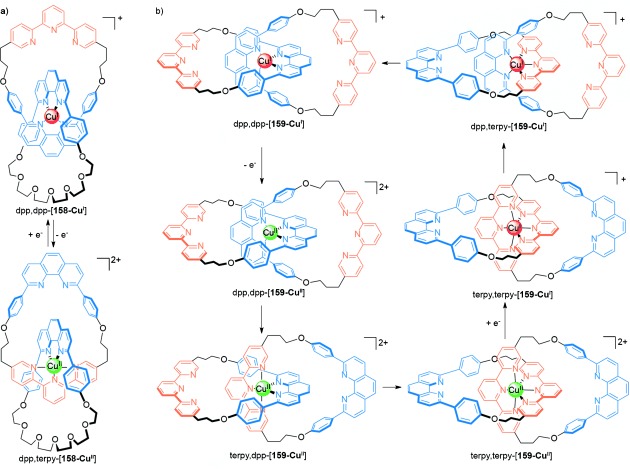
a) Sauvage's switchable heterocatenate 158.[143] b) Oxidation-state-controlled switching of [2]catenate 159 between three distinct co-conformations.[144]

Sauvage and co-workers demonstrated both electrochemical and photochemical control over ring motions in hetero-[2]catenate **158** (Scheme 46 a).[143] Although many metal–ligand interactions can be rather kinetically stable, the difference in the preferred coordination geometries of different oxidation states can be exploited to bring about configurational switching in [2]catenanes. Oxidation of the metal center in dpp,dpp-[**158-Cu^I^**] (by either chemical or electrochemical means) reverses the order of preference for coordination numbers (the preferred order for Cu^II^ is 6>5>4). Thus, a configurational change to the five-coordinate state obtained in dpp,terpy-[**158-Cu^II^**] is observed. The kinetics of intercomponent motion in the switching process are relatively slow due to the generation of a metastable dpp,dpp-[**158-Cu^II^**] state following oxidation. Whilst the process can be reversed on reduction to regenerate dpp,dpp-[**158-Cu^I^**], the formation of a metastable dpp,terpy-[**158-Cu^I^**] state makes the switching slow once again.

The related homo-[2]catenate **159** (Scheme 46 b), in which each ring contains a bidentate dpp unit and a tridentate terpy site, exhibits more complicated behavior.[144] In dpp,dpp-[**159-Cu^I^**] the copper ion coordinates to the two dpp units in the usual tetrahedral arrangement. Following oxidation of the metal cation, circumrotation of the rings proceeds to give the preferred hexacoordinated species terpy,terpy-[**159-Cu^II^**]. It was demonstrated that this process occurs by the revolution of one ring with respect to the other to give an intermediate five-coordinate species (terpy,dpp-[**159-Cu^II^**]).[144] In comparison to many related coordination complexes, the process is relatively fast, with the ligand rearrangement occurring on the timescale of tens of seconds. The process is completely reversible, via the same five-coordinate geometry, on reduction to Cu^I^ (that is, via dpp,terpy-[**159-Cu^I^**]).

**Scheme 47:**
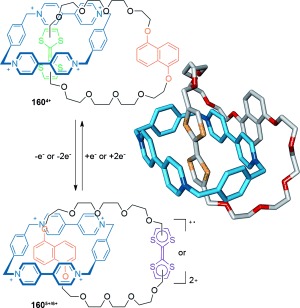
Stoddart's co-conformational switching of a [2]catenane mediated by the relative strengths of intermacrocycle electron-rich/poor aromatic stacking interactions.[145]

The Stoddart group demonstrated the co-conformational switching of [2]catenane **160** containing an electron-poor cyclobisparaquat(*p*-phenylene) (CBPQT^4+^) macrocycle, and an electron-rich crown ether macrocycle (Scheme 47).[145] In the initial **160^4+^** state, the tetrathiafulvalene (TTF) unit in the crown ether macrocycle is the more electron-rich motif, and is predominantly situated within the cavity of the tetracationic cyclophane. However, following TTF oxidation, either through chemical or electrochemical means, the dioxynaphthalene unit becomes the more electron-rich motif and the structure switches to accommodate that unit within the crown ether cavity (**160^5+^**/**160^6+^**). The process can be reversed through reduction of the TTF back to its neutral state.

**Scheme 48:**
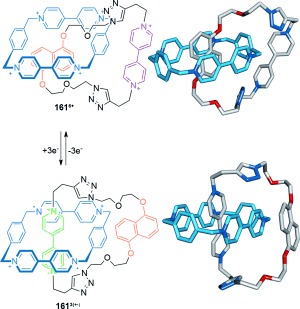
Stoddart's reversible switching in a [2]catenane by alternating between electron-rich/poor aromatic stacking interactions and radical-pairing interactions as dominant intermacrocycle forces, after bipyridinium reduction and oxidation, respectively.[146]

A related [2]catenane features co-conformational switching mediated by electrostatic π–π interactions and radical pairing as the dominant intermacrocycle forces (Scheme 48).[146] The CBPQT^4+^ macrocycle resides over a dioxynaphthalene unit in catenane **161^6+^**. On reduction of bipyridinium (bipy^2+^) units to bipy^+^**^.^**, radical pairing interactions change the predominant structure to catenane **161^3(+.)^**. Oxidation regenerates **161^6+^** and reverses the switching process.

**Scheme 49:**
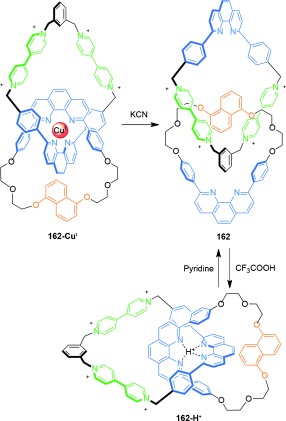
Sauvage's pH-induced switching between co-conformations following demetalation of a Cu^I^-template-directed [2]catenane.[147]

The pH-induced switching of a [2]catenane between two well-defined co-conformations has been demonstrated by Sauvage and co-workers (Scheme 49).[147] Reversible switching of the catenanes co-conformations between **162** and **162-H^+^** is achieved by protonation and deprotonation of the catenane respectively.

**Scheme 50:**
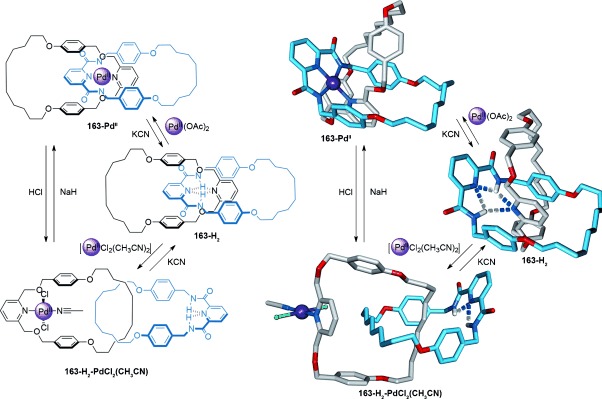
A three-state switching process between two co-conformations for a [2]catenane utilizing variable coordination modes of suitable Pd^II^ cations.[148]

A combination of metal coordination/demetalation and hydrogen bonding results in reversible switching of [2]catenane **163-Pd^II^** (Scheme 50).[148] In both **163-Pd^II^** and **163-H_2_**, the pyridine groups of each macrocycle are held in proximity through their involvement in metal coordination and hydrogen bonding, respectively. However, addition of [PdCl_2_(CH_3_CN)_2_] to **163-H_2_** generates **163-H_2_-PdCl_2_(CH_3_CN)** which has a preferred co-conformation in which one ring is rotated 180° with respect to the other. The switching process can be reversed through deprotonation of the amide nitrogen atoms with NaH, or by demetalation and reintroduction of a basic Pd^II^ salt.

**Scheme 51:**
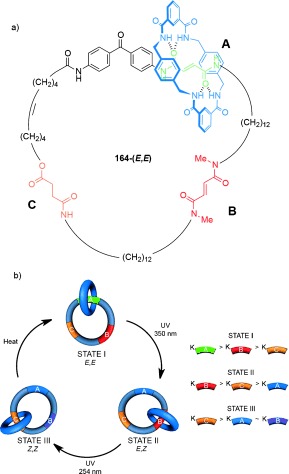
A [2]catenane in which modifying the binding affinity of three different stations to a benzylamide macrocycle allows switching between three discrete co-conformations. a) Structure of [2]catenane 164-(*E*,*E*). b) Illustration of the switching process.[102a

Nondirectional switching between three different co-conformations can be brought about with [2]catenane **164** (Scheme 51 a).[102a Three “stations” are incorporated into the larger macrocycle ring: A (green), B (red), and C (yellow). Each station has a different binding affinity for the smaller benzylic amide macrocycle. The affinity of fumaramide groups at A and B can be drastically reduced by photoisomerization to the maleamide (*Z*) form, which occurs at different wavelengths for the two stations because of the proximity of the benzophenone group to A.

In its initial state (*E*,*E*), the binding affinity of the stations to the small macrocycle is in the order A>B>C, and thus the benzylic amide macrocycle is predominantly located over station A. Following *E* to *Z* isomerization of A, the relative affinities change to B>C>A and the small macrocycle moves predominantly to station B (State I to State II, Scheme 51 b). A further change in the position of the benzylic amide macrocycle occurs upon isomerization of station B, thereby locating the macrocycle preferentially at station C (State II to State III, Scheme 51 b), with station binding affinity now in the order C>A≈B. Re-isomerizations of A and B to fumaramide groups restores the initial station binding affinity order of A>B>C, and returns the benzylic amide macrocycle to its original site.

Switching in other catenanes has been achieved using anion binding[149] and photochemical stimuli.[150]

### 5.2. Catenanes as Rotary Motors

**Scheme 52:**
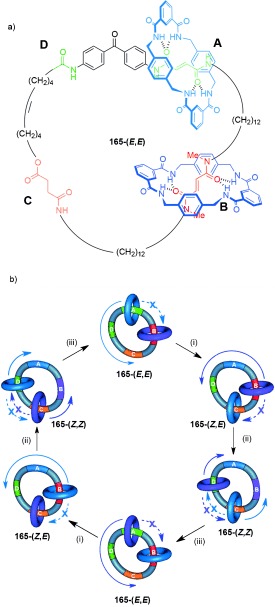
A [3]catenane rotary motor. a) Structure of [3]catenane 165-(*E*,*E*).[102a b) Illustration of the directional switching process. Conditions: i) UV (350 nm); ii) UV (254 nm); iii) heat.

Introducing kinetic barriers to restrict the pathways of Brownian motion available to a macrocycle in a catenane can be used to produce net-directional rotary motion and a molecular motor. [3]catenane **165** (Scheme 52 a)[102a differs from [2]catenane **164** (Scheme 51 a) only by the addition of a further benzylic amide macrocycle, and it features the same tunable station-binding affinities to control the position of the smaller macrocycles. The isolated amide group (dark green) acts as a fourth binding station, D. The station binding affinities for the benzylic amide macrocycles follow the order A>B>C>D in **165-(*E***,***E*****)**, and the two macrocycles are accordingly positioned primarily over the A and B stations.

Following photoisomerization of station A, the blue macrocycle moves from station A to C (*K*_B_>*K*_C_>*K*_D_>*K*_A_). Importantly, translocation of the ring can only occur in one direction around the larger ring (anticlockwise from Scheme 52 b) because of the presence of the second macrocycle on station B. Likewise, on *E* to *Z* isomerization of station B, translation of the purple macrocycle can only proceed in one direction (anticlockwise) to station D (*K*_C_>*K*_D_>*K*_A_≈*K*_B_). Hence, the small rings move in a “follow-the-leader” process directionally around the larger ring. The entire reaction sequence must be repeated for a full directional 360° rotation of each macrocycle to occur.

**Scheme 53:**
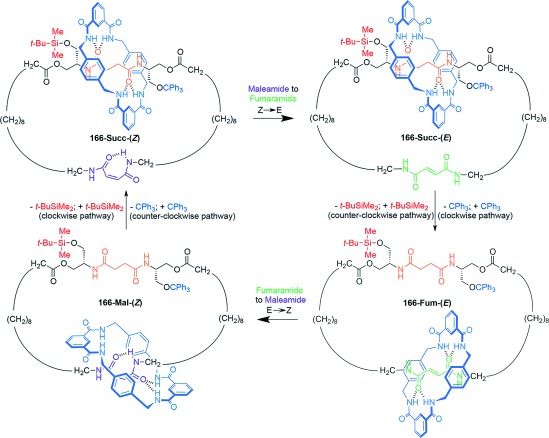
Cleavage and switching sequences in rotary motor [2]catenane 166, which is capable of directional and reversible circumrotation.[102b

Selective rotation in either direction is possible using [2]catenane **166** (Scheme 53).[102b In this case, the route of travel of the smaller ring around the larger one is determined by the order in which blocking groups either side of the succinamide binding site are released. Starting from **166-Succ-(*Z*)**, isomerization of the maleamide station (purple) to its fumaramide form (green) makes it the preferred binding station for the benzylic amide macrocycle. Removing one of the blocking groups (*t*BuSiMe_2_ for counterclockwise rotation; CPh_3_ for clockwise rotation) allows for directional movement of the ring to give **166-Fum-(*E*)**. The fumaramide station can then be isomerized to the maleamide form, thereby creating a thermodynamic driving force for movement of the macrocycle back to the succinamide station in **166-Mal-(*Z*)**. Removal of the blocking group opposite to that used in the first change (i.e. CPh_3_ for counterclockwise rotation; *t*BuSiMe_2_ for clockwise rotation) results in a full 360° directional rotation of the benzylic amide macrocycle around the large macrocycle.

### 5.3. Catenanes as Selective Hosts for Anions and Cations

As is apparent from this Review, the driving force for catenane formation is often the attractive noncovalent interactions between the components and/or a template. These interactions often “live on” after the interlocked structure is assembled, and any cavity in the 3D space formed by such a catenane then has functional groups in precise positions that are often well-matched to bind substrates with high specificity. These features have proved particularly useful in the case of catenanes synthesized with anion templates, with the resulting catenanes in some cases being able to selectively bind particular anions.[151] This, in turn, has led to catenanes being modified to signal binding events photo- or electrochemically.

**Scheme 54:**
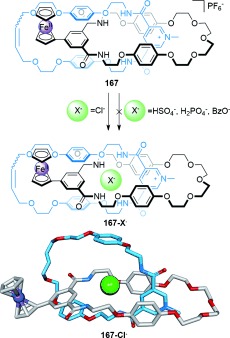
Beer's incorporation of a ferrocene motif into a catenane allows electrochemical sensing of anion binding in 167, which was found to selectively bind chloride anions. The corresponding non-interlocked macrocycles of 167 were found to display binding selectivity dictated by the basicity of the anions (BzO^−^>H_2_PO_4_^−^>Cl^−^>HSO_4_^−^).[152]

Beer and co-workers used ferrocene-derivatized [2]catenane **167** (Scheme 54) to selectively complex chloride anions over oxoanions and electrochemically report the binding.[152] The binding of chloride facilitates the oxidation of the ferrocene to ferrocinium and can be detected by cyclic voltammetry. The X-ray structure of **167-Cl^−^** suggests that the selectivity in anion binding is probably due to the catenane cavity being too small to accommodate larger anions.

**Scheme 55:**
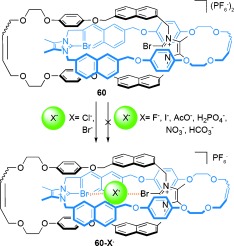
Beer's selective binding of chloride and bromide anions detected by optical sensing of their influence on the naphthalene emission spectra.[80]

An optical response occurs upon anion binding to [2]catenane **60** (Scheme 55, the synthesis is outlined in Section 2.1.4).[80] Chloride or bromide anions significantly decrease the intensity of the naphthalene emission band (309 nm) concomitant with the appearance of a new band (445 nm). No changes in the emission spectrum were detected with a range of alternative anions. Jeong and co-workers have also used fluorescence to measure selective chloride binding using a [2]catenane containing indolocarbazole fluorophores.[153]

**Scheme 56:**
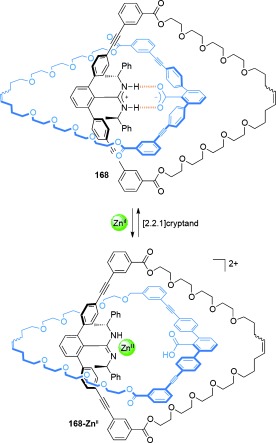
Yashima's cation-sensing catenane which undergoes a switch from a “locked” state to one in which macrocycles can freely rotate.[154]

[2]Catenane **168** has been employed by Yashima and co-workers[154] to detect cation binding through changes in circular dichroism (CD; Scheme 56). The chiral *R*-phenylethyl substituents on the amidinium side of the salt bridge induce twisting of the *m-*terphenyl motifs in both macrocycles of the catenane, thereby creating distinct Cotton effects in the CD spectrum. On the addition of Zn(ClO_4_)_2_, the salt bridge is disrupted through Zn^II^ coordination, which results in significant changes in the CD spectrum.

## 6. Catenane Linkages Incorporated into Polymer Chains, Materials, and Attached to Surfaces

Given the high cost of introducing new chemical building blocks, it seems likely that future generations of commercial polymers will be derived from assembling existing cheap monomers in new ways. Incorporating catenanes into a polymer backbone offers the possibility of introducing flexible, mobile, or switchable linkages which could influence the rheological,[155] mechanical,[156] and dynamic[157] properties of the resulting material. Harnessing the coordinated motion of the components of catenanes in ordered arrays could be useful for solid-state devices, and could arise from embedding catenanes onto or within a support material, such as a metal–organic framework (MOF), or on a functionalized surface.

### 6.1. [2]Catenanes in Polymers

#### 6.1.1. [2]Catenanes as Main-Chain Linkages

**Figure 9:**
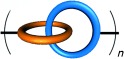
A main-chain poly[2]catenane.

**Scheme 57:**
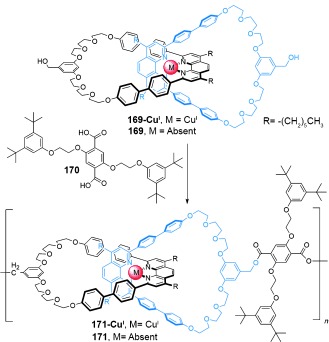
Geert and Sauvage's synthesis of main-chain poly[2]catenanes incorporating dpp-Cu^I^ catenanes or their demetalated analogues.[158], [159]

Main-chain poly[2]catenanes consist of [2]catenanes connected in a linear manner by covalent bonds (Figure 9). Polycondensation of catenate **169-Cu^I^**, or the corresponding metal-free catenand **169**, with dicarboxylic acid **170** afforded polymers **171-Cu^I^** and **171**,[158] although the degree of polymerization in these systems is probably modest (Scheme 57).[159] Related poly[2]catenanes based on Cu^I^-dpp [2]catenane motifs have also been reported by Shimada et al.[160]

**Scheme 58:**
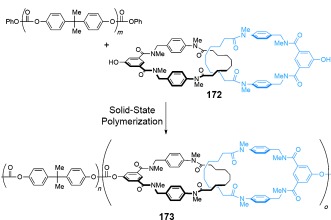
The solid-state polymerization of [2]catenane monomer 172 and bisphenol A polycarbonate oligomers to generate a main-chain poly[2]catenane copolymer.[161a

To probe the influence of mechanical links in polymer chains, benzylic amide [2]catenanes have been used to dope commercial polymers (polycarbonate and poly(ethylene terephthalate)).[161] The phenol groups of catenane **172** provide sites for transesterification into the polymers, whilst methylation of the amide nitrogen atoms removes the possibility of intercomponent hydrogen bonding and allows relatively free movement of the rings for amplification of any physical properties endowed by the mechanical bond (Scheme 53). Solid-state polymerization of catenane **172** and bisphenol A polycarbonate oligomers (Scheme 58) was conducted to generate copolymers consisting of 10, 20, or 30 wt % catenane linkages.[161a Homogeneous dispersion of the [2]catenane was observed at low levels of incorporation (10 wt %), but heterogeneity probably occurs at higher levels of incorporation. Incorporating the catenane links had a modest effect on the glass transition temperature (*T*_g_) of the resulting polymer. Hagiwara et al. have utilized alternatively functionalized derivatives of catenane **172** for polymerization with rigid dialkyne complexes through Sonogashira cross coupling[162] and CuAAC “click” reactions.[163] Other examples of poly[2]catenanes have also been prepared.[164]

#### 6.1.2. Catenanes as Polymer Side Chains

**Figure 10:**
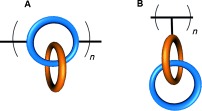
Side chain poly[2]catenanes in which (A) a single macrocycle forms part of the polymer main chain or (B) the entire catenane is a pendent group.

Unlike main-chain poly[2]catenanes, creating polymers with catenane side chains requires functionalization of only a single macrocycle within the catenane. As the catenane′s mechanical bond no longer constitutes part of the polymer backbone (Figure 10), it is expected to induce different properties in the resulting material. Polymers with catenane side chains have been synthesized by polymerization across a single macrocycle of the catenane,[165] or grafting of catenanes onto a preformed polymer.[166]

**Scheme 59:**
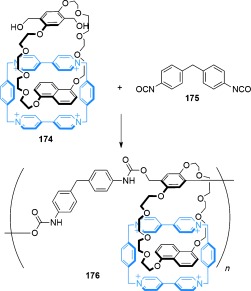
Stoddart's side-chain poly[2]catenane in which a single macrocycle of the [2]catenane constitutes part of the polymer backbone.[165a

Side-chain poly[2]catenane **176** was prepared by treating diol catenane **174** with bis(4-isocyanatophenyl)methane (**175**, Scheme 59).[165a A pendent poly[2]catenane was prepared by Simone and Swager[165b by electrochemical polymerization.

**Scheme 60:**
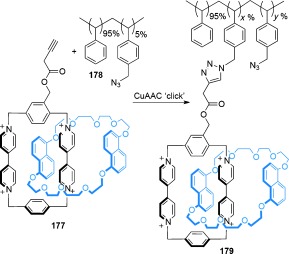
Bria's side-chain [2]catenane polymer.[166]

An alternative strategy to pendent poly[2]catenanes was demonstrated by Bria et al.[166] Functionalization of [2]catenane monomer **177** (Scheme 60) with a single alkyne group allows its incorporation into copolymer **178** through CuAAC “click” reactions to give **179**.

#### 6.1.3. Catenanes Incorporated into Metal–Organic Frameworks

**Scheme 61:**
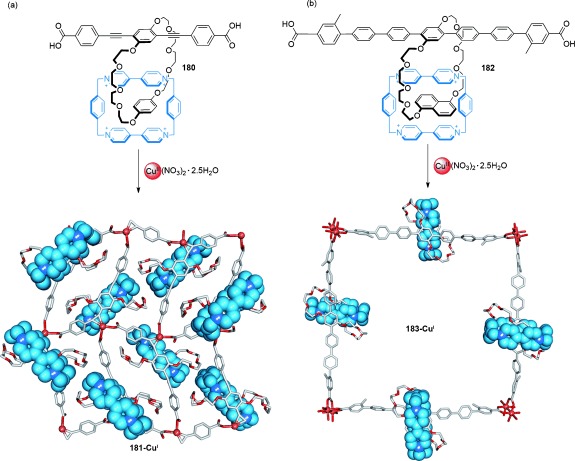
Stoddart and Yaghi's synthesis of metal–organic frameworks containing [2]catenane organic struts.[168], [169] The interlocked macrocycles which do not form part of the MOF backbone have been colored blue, and represented as space-filled structures for clarity.

The highly ordered arrangement of molecules in crystalline solids, combined with the space available for component movements, has led to metal–organic frameworks (MOFs) being investigated as scaffolds for interlocked molecules. Whilst rotaxanes have been incorporated into different types of MOFs,[167] catenanes present a significant challenge because of their size and shape. Nevertheless, MOFs containing [2]catenanes have been described by Stoddart, Yaghi, and co-workers.[168] The catenane-bearing molecular strut **180** forms a 2D array after heating with Cu(NO_3_)_2_⋅2.5 H_2_O (Scheme 61 a). Within the MOF, each Cu^I^ cation coordinates to the carboxylate groups of two struts, and to the acetylene group of an additional **180** in an η^2^ fashion. Accommodating such large molecules requires the network to form an alternating sequence of catenane orientations positioned above and below the plane of the 2D sheet, with further long-range-ordering observed between the stacked arrays. A more regular 2D MOF was formed using a related catenane incorporated into a longer coordinating strut (Scheme 61 b),[169] whilst a related 3D MOF has also been reported.[170]

### 6.2. Catenanes Attached to Surfaces

**Figure 11:**
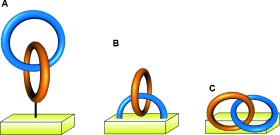
Three different forms of [2]catenanes attached to surfaces.

Attaching catenanes to surfaces is an alternative approach to obtaining ordered arrays of interlocked molecules. Catenanes can be fastened to surfaces in different ways: 1) attached through a tether (Figure 11, type A); 2) chemisorbed such that the surface forms an intrinsic part of one ring (Figure 11, type B); or 3) physisorbed (Figure 11, type C).[171]

**Scheme 62:**
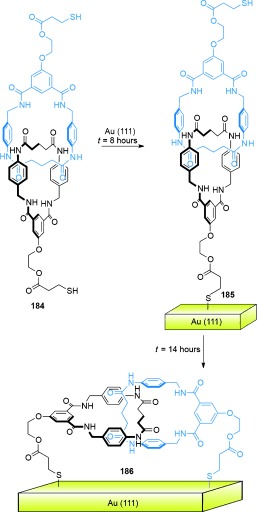
A thiol-functionalized [2]catenane tethered to a Au(111) surface.[172]

The process of tethering a [2]catenane to a gold surface is illustrated with dithiol [2]catenane **184** (Scheme 62).[172] X-Ray photoelectron spectroscopy (XPS) indicates that **184** initially forms a monolayer in which the catenanes are arranged perpendicular to the surface, chemisorbed through only one of the two thiol groups. Given enough time, however, (14 h in the case of **184**) the catenane finds a lower energy co-conformation in which both thiol groups bind to the gold, thereby holding the catenanes parallel to the surface.

**Scheme 63:**
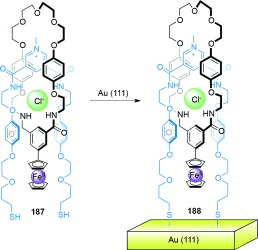
Beer's chloride-containing pseudorotaxane which forms a [2]catenane with a Au(111) surface.[152]

Beer′s chloride-binding catenane with an electrochemically active ferrocene reporting group (see Section 5.3) has also been adapted for incorporation onto a surface.[152] Exposure of the dithiol pseudorotaxane complex **187** (Scheme 63) to a Au(111) surface led to formation of a self-assembled monolayer (**188**) after 8 h that is effectively a [2]catenane in which the Au(111) surface forms part of one ring. This type of strategy for forming [2]catenanes at a surface was originally introduced by Gokel, Kaifer, and co-workers.[173] Other catenane monolayers have been described by the Sauvage group.[174]

**Scheme 64:**
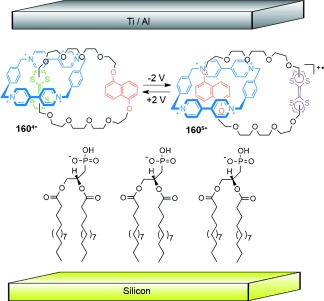
Stoddart's switchable [2]catenane layered between electrodes.[175]

Co-conformational switching has also been demonstrated in a mixed Langmuir monolayer of [2]catenane **160** and phospholipid dimyristoylphosphatidic acid (DMPA) sandwiched between an Si electrode and a Ti/Al electrode (Scheme 64, see also Section 5.1.1).[175] Application of a −2 V or +2 V bias to the system results in oxidation of **160^4+^** or partial reduction of **160^5+^**, respectively. A significant change in the junction resistance was observed on cycling between **160^4+^** and **160^5+^**.

**Scheme 65:**
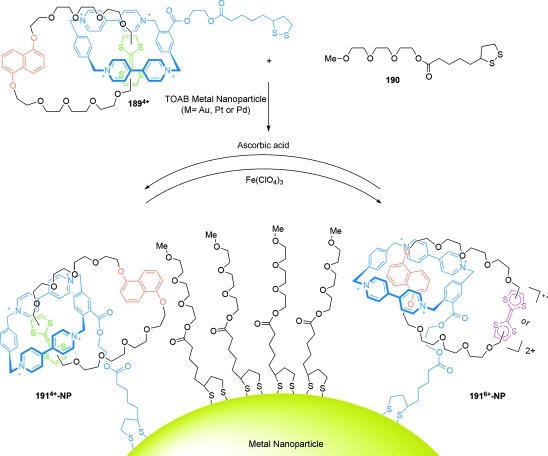
Stoddart's switchable [2]catenane tethered to a metal nanoparticle. TOAB=tetraoctylammonium bromide.[176]

Co-conformational switching can occur with catenanes tethered to a surface. Stoddart and co-workers[176] demonstrated that the TTF-based catenane **189^4+^** (Scheme 65) still undergoes switching when chemisorbed on metal nanoparticles (Au, Pd, or Pt). Reversible oxidation and reduction of the surface-grafted catenanes between **189^4+^-NP** and **189^6+^-NP** was achieved with Fe(ClO_4_)_3_ and ascorbic acid, respectively.

Benzylic amide catenanes have been extensively studied as chemisorbed and physisorbed thin films on various surfaces. Well-ordered films of catenanes such as **57** can be grown by sublimation under ultrahigh vacuum, and catenanes of the first monolayer were chemisorbed on a Au surface through some of their carbonyl groups. Their electronic and vibrational structures have been characterized by XPS and high-resolution electron energy loss spectroscopy (HREELS) as well as by infrared reflection absorption spectroscopy.[177] Physisorbed surface catenanes have also been demonstrated by the Sauvage group.[178]

Despite these successes, introducing catenanes into polymers or assembling them into ordered arrays on surfaces or within crystalline solids remains a very significant challenge. Exploiting the dynamic properties of catenanes―—inherent flexibility, rotation, or switching of the relative positions of the rings―—in such environments has yet to be achieved in any genuinely useful manner.

## 7. Conclusions and Outlook

### The End of an Era…=

The template synthesis of catenanes is unquestionably one of the great triumphs of synthetic supramolecular chemistry. It provides the means through which chemists can today routinely make Hopf link catenanes in one step, in high yields, from readily available building blocks. Such a situation was simply unimaginable fifty years ago in an era when there was little or no understanding of template synthesis, host–guest chemistry, self-assembly processes, or the utilization of noncovalent interactions, nor any of the highly effective modern tools for covalent-bond formation. Indeed, the success of interlocked molecule synthesis is testament to just how dramatically chemistry can progress. The brilliance of Schill and Lüttringhaus demonstrated how chemists could design synthetic pathways that feature spatial awareness in all three dimensions, a necessity to bring about the controlled crossing of molecular strands and/or the threading of an organic chain through a macrocycle. Sauvage′s great contribution was to realize that the synthesis of such structures could be greatly facilitated by using the preferred coordination geometry of transition-metal ions to assemble and orientate the molecular building blocks, whilst Stoddart′s (as well as pioneering much of the synthetic chemistry on catenanes and rotaxanes in the 1990s) was to recognize the potential of mechanically bonded molecules as architectures for molecular machines. In articulating that vision, he provided an incentive for a generation of chemists to use their imagination and creativity to come up with ever more inventive and successful routes to interlocked molecules.

The resulting accomplishments—in template syntheses that exploit metal coordination, hydrogen bonding, aromatic stacking interactions, the hydrophobic effect, halogen bonding, and ever-improving methods of covalent capture such as active template synthesis, dynamic covalent chemistry, olefin metathesis, and “click” chemistries—―mean that in 2015 the problem of how to form Hopf link [2]catenanes is effectively solved.

### …=.and the Start of a New One

The rest of the field of chemical topology remains almost completely virgin territory. The synthesis of catenanes is straightforward only for the simplest Hopf link topology. Only a handful of the other links listed in Figure 2 (and there are countless other possibilities)[21] have been prepared thus far (Solomon links, Borromean rings, and one Star of David catenane)[179] and the dynamics of the components in those topologies, and how to control or exploit them, remain unknown. Whilst it is often suggested that given enough time and resources today′s chemists can make almost any molecule,[180] that is simply not true of topologically complex structures. No general method yet exists to make catenanes more complicated than a Hopf link, nor is there any strategy for controlling stereochemistry (over or under) at crossing points (such control is necessary to make all but the most trivial links and knots), nor methods to effectively incorporate catenanes into materials (polymers, MOFs, or at surfaces and interfaces) and exploit their interlocked architectures and dynamics. These are major unsolved problems―—in synthesis, design, and application—―that currently preclude the exploration and exploitation of a vast region of chemical space. Indeed, many of the unmet challenges in chemical topology today seem even more perplexing than the problems faced by the pioneers of catenane chemistry fifty years ago

Biology shows us time and again that the organization of matter with topological complexity at the molecular level offers tremendous potential for achieving novel molecular and material properties (strength, lightweight, flexibility, dynamics). Chemists can uncover the ways and means to tap into this hitherto unrealized wealth of form and function if they can make inroads into what is, perhaps, one of the last great unexplored frontiers of synthetic chemistry. Exactly what the next half-century of catenane research will uncover is impossible to foresee, but surely it will be as rich, diverse, and full of extraordinary surprises as the first fifty years.
